# Research progress of organic liquid electrolyte for sodium ion battery

**DOI:** 10.3389/fchem.2023.1253959

**Published:** 2023-09-12

**Authors:** Jia Zhang, Jianwei Li, Huaiyou Wang, Min Wang

**Affiliations:** ^1^ Key Laboratory of Comprehensive and Highly Efficient Utilization of Salt Lake Resources, Qinghai Institute of Salt Lakes, Chinese Academy of Sciences, Xining, China; ^2^ Key Laboratory of Salt Lake Resources Chemistry of Qinghai Province, Xining, China; ^3^ School of Chemical Sciences, University of Chinese Academy of Sciences, Beijing, China

**Keywords:** sodium ion battery, organic liquid electrolyte, cathode, anode, solid electrolyte interphase (SEI)

## Abstract

Electrochemical energy storage technology has attracted widespread attention due to its low cost and high energy efficiency in recent years. Among the electrochemical energy storage technologies, sodium ion batteries have been widely focused due to the advantages of abundant sodium resources, low price and similar properties to lithium. In the basic structure of sodium ion battery, the electrolyte determines the electrochemical window and electrochemical performance of the battery, controls the properties of the electrode/electrolyte interface, and affects the safety of sodium ion batteries. Organic liquid electrolytes are widely used because of their low viscosity, high dielectric constant, and compatibility with common cathodes and anodes. However, there are problems such as low oxidation potential, high flammability and safety hazards. Therefore, the development of novel, low-cost, high-performance organic liquid electrolytes is essential for the commercial application of sodium ion batteries. In this paper, the basic requirements and main classifications of organic liquid electrolytes for sodium ion batteries have been introduced. The current research status of organic liquid electrolytes for sodium ion batteries has been highlighted, including compatibility with various types of electrodes and electrochemical properties such as multiplicative performance and cycling performance of electrode materials in electrolytes. The composition, formation mechanism and regulation strategies of interfacial films have been explained. Finally, the development trends of sodium ion battery electrolytes in terms of compatibility with materials, safety and stable interfacial film formation are pointed out in the future.

## 1 Introduction

Fossil fuels are the most widely used energy source in the world, however, its non-renewable and unsustainable nature makes it increasingly depleted, and the burning of fossil fuels causes a series of problems such as global warming and atmospheric pollution (Gao et al., 2020). Therefore, the development of renewable energy is becoming more and more important. However, the conversion of renewable energy into electrical energy is variable, intermittent and unpredictable (Cao et al., 2013), so it is necessary to develop energy storage technology to realize the scale of grid-connected storage of electrical energy and guarantee the continuous and stable electricity supply to users (Dunn et al., 2011). Electrochemical energy storage technologies have received much attention due to their high energy efficiency and high power density (Dunn et al., 2011; Yang et al., 2011). However, few, if any, electrochemical grid-scale energy storage technologies, when implemented at system-level, are currently “low cost” and “long lifetime”, especially when compared to standard natural gas peaker plants. Electrochemical energy storage technologies certainly have the potential to become “low cost” and “long lifetime” in future. More research is needed to achieve this goal and make it economically attractive compared to fossil fuels (Larcher and Tarascon, 2015).

As a mainstream electrochemical energy storage technology, lithium-ion batteries are widely used in our life by virtue of their high energy density and long cycle life. Additionally, the manufacturing scale of lithium-ion batteries continues to expand, which will inevitably cause huge consumption of lithium resources and soaring prices (Li et al., 2017). The element lithium is not abundant and unevenly distributed in the earth’s crust (Pan et al., 2013), and in China, it is 70% of the lithium used depends on imports (RONG et al., 2020). In order to avoid the problem of “neck” due to the shortage of resources, it is necessary to develop an energy storage technology that is comparable to lithium-ion batteries. In the periodic table, sodium and lithium are metal elements in the same group and posess similar physical and chemical properties. The earth is rich in sodium, with an elemental content of about 23,000 ppm (lithium content is only about 17 ppm), making it sixth place in terms of abundance. Sodium is distributed all over the world, completely free from resource and geographical constraints. Therefore, sodium-ion batteries have a greater promise than lithium-ion batteries. The research of sodium ion battery can mitigate resource problem of new energy battery development caused by the shortage of lithium resources. Sodium ion batteries (SIBs) include sodium-sulfur batteries, sodium-salt batteries (ZEBRA batteries), sodium-air batteries, organic-based sodium-ion batteries and aqueous-based sodium-ion batteries. Among them, sodium-sulfur batteries are based on the electrochemical reaction of sodium and sulfur to generate sodium polysulfide, and are characterized by high power and energy density, temperature stability, and low cost because of the abundant cost of its raw materials, and have already achieved large-scale production (Wen et al., 2008). ZEBRA batteries use common salt and nickel as the raw materials for the electrodes, and are combined with ceramic electrolyte and molten salt. This combination provides battery systems with high specific energy and power. ZEBRA battery technology has been industrialized for all types of electric cars and hybrid electric buses (Dustmann, 2004). Sodium-air batteries, organic sodium-ion batteries and aqueous sodium-ion batteries are still in the research phase. Sodium ion battery also has the advantages of low cost, excellent fast charging and low temperature performance, good safety performance, *etc.* The manufacturing of sodium ion battery can follow the production process and equipment of existing lithium ion battery, which is considered as one of the transformative technologies in the field of large-scale energy storage, and its industrialization prospect is quite optimistic and has important economic and strategic significance (Fang et al., 2018; Lu et al., 2018; ZHOU et al., 2020). Therefore, sodium ion batteries are called the “rising star” of the energy storage field.

Sodium ion battery is mainly composed of three parts: cathode, anode and electrolyte. The working principle is similar to that of lithium-ion battery. During the charging process, the cathode material loses electrons in the oxidation reaction and electrons move from the external circuit to the anode, while sodium ions (Na^+^) are removed from the cathode and enter the electrolyte, then migrate through the electrolyte to the vicinity of the anode and finally embedded in the anode material; during the discharge process, the anode electrode material loses electrons in the oxidation reaction and electrons move through the external circuit to the cathode, while Na^+^ is removed from the anode material and embedded in the anode material. In the process of discharge, the anode material loses electrons through oxidation and electrons move to the cathode through the external circuit (Pan et al., 2013). As one of the main components of sodium ion battery, electrolyte has an important role in conducting ions and participating in the redox reaction of cathode and anode (ZHU et al., 2016). Electrolyte is the “bridge” connecting cathode and anode. The performance of the electrolyte directly affects the performance of sodium ion batteries. During the charging and discharging process, the electrolyte itself decomposes or reacts with the electrode material to form an interface. The interfacial film on the anode is called the solid electrolyte interphase (SEI), and the interfacial film on the cathode is called the cathodic electrolyte interphase (CEI). CEI and SEI largely determine the electrochemical performance of the battery system (Lin et al., 2019). At present, sodium ion battery electrolyte system mainly includes aqueous electrolyte and non-aqueous electrolyte. Non-aqueous electrolyte contains organic liquid electrolyte and solid electrolyte. The recyclability of aqueous electrolyte is excellent but its electrochemical window is narrow and the overall energy density is low (Liu S. et al., 2018). Solid electrolytes generally have higher impedance and polarization, leading to a decrease in battery capacity. Moreover, the solid electrolyte itself has a narrow electrochemical window, and the mismatch with high-voltage electrodes will cause side reactions, leading to the deterioration of battery cycling performance (Hu et al., 2020; Gao et al., 2021). In a comprehensive comparison, organic liquid electrolytes have good properties, such as electrochemical stability within a certain electrochemical window, sufficiently high ionic conductivity, and good compatibility with various electrode materials. Therefore, organic liquid electrolytes are the most promising electrolytes for sodium ion batteries in practical applications.

This paper summarizes the research progress of organic liquid electrolytes for sodium ion batteries by discussing the basic requirements and composition of organic electrolytes for sodium ion batteries, the current research status of organic liquid electrolytes, the composition and requirements of the interface between electrolytes and electrodes and the regulation strategies. Finally, the performance of organic electrolyte and the nature of interfacial film are synthesized, and some suggestions on the future development trend of organic electrolyte for sodium ion batteries are proposed, in order to provide some help to the research of sodium ion battery electrolyte and sodium ion battery energy storage science and technology.

## 2 Components and basic requirements of organic liquid electrolytes

**FIGURE 1:**
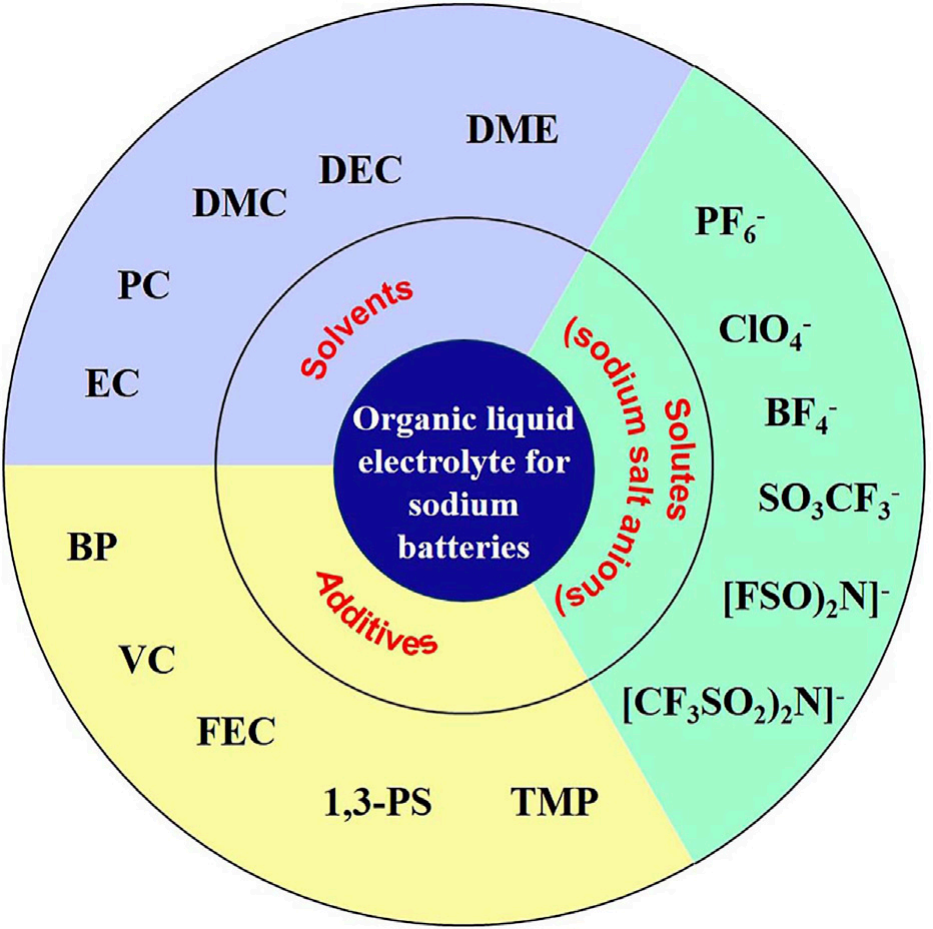
Main components of sodium ion battery electrolyte (Hu et al., 2020).

As a bridge connecting cathode and anode, the electrolyte assumes the role of transporting ions between cathode and anode and is an important part of the battery, whichplays a vital role in the performance of the battery in terms of multiplicity, cycle life, safety and self-discharge. Organic liquid electrolyte is also customarily called organic electrolyte. Electrolyte is mainly composed of solvent, solute and additives (Figure 1), which together determine the properties of electrolyte.

### 2.1 Solvent

Solvent is one of the important components of organic liquid electrolytes. Electrolyte solvents need to satisfy the most basic conditions such as stability, non-toxicity and cheapness. Besides, the electrolyte solvent should have a wide electrochemical stability window, sufficient sodium salt solubility, high dielectric constant, low viscosity and wide liquid range (i.e., low melting point and high boiling point). The solvent should also maintain electrochemical stability or promote the formation of a high-quality passivation layer during cell operation. However, these different and sometimes conflicting requirements for the same solvent are difficult to be met by a single solvent, and therefore multiple solvents are often used in combination. The main solvents currently used in sodium ion batteries are ester solvents and ether solvents. These two types of organic solvents have provided excellent performance in battery applications (Fenton, 1973; Armand et al., 2009; Monti et al., 2014; Hong et al., 2015; Ponrouch et al., 2015; Niu et al., 2019; Wang et al., 2020).

Ester solvents are a more commonly used class of solvents, especially cyclic [propylene carbonate (PC) and ethylene carbonate (EC)] and chain [dimethyl carbonate (DMC), diethyl carbonate (DEC) and methyl ethyl carbonate (EMC)] carbonates are most commonly used, and electrolytes of carbonate solvents tend to have the advantages of high ionic conductivity and good oxidation resistance (Ponrouch et al., 2012). The relevant physical and chemical properties of carbonate solvents are shown in Table 1. The dielectric constant of ether solvents is much lower than that of cyclic carbonates, but higher than that of chain carbonates. The resistance to oxidation is relatively poor, and they tend to decompose at high voltages. However, ether solvents generate thinner SEI on anode with higher initial Coulomb efficiency, and ether solvents are more compatible with anode such as metallic sodium and can co-embed graphite with sodium ions and show good reversibility, making graphite that cannot be embedded with sodium in ester solvents can be used as anode in this solvent system (Dubois et al., 1997). In the actual application process, the use of two or even a variety of solvent mix is a more common method (Komaba et al., 2011b), the ratio of different solvents is controlled, and the advantages of multiple solvents are integrated to maximize the performance of electrolytes.

**TABLE 1:** Physical and chemical properties of some commonly used organic solvents (Xu, 2004; Kamath et al., 2014; Vignarooban et al., 2016; Chen et al., 2019; Wang M. et al., 2022).

Solvents	Density/g·cm^-3^	Boiling point/°C	Melting point/°C	Viscosity/10^−3^ Pa s 25°C	Dielectric constant/F m^-1^ 25°C	HOMO/eV	LUMO/eV
DMC	1.063	91	4.6	0.59	3.107	−0.2488	−0.0091
DEC	0.969	126	−74.3	0.75	2.805	−0.2426	−0.0036
EC	1.321	248	36.4	2.1	89.78	−0.2585	−0.0177
PC	1.200	242	−48.8	2.53	64.92	−0.2547	−0.0149
EMC	1.006	110	−53.0	0.65	2.95	−0.2557	−0.0062

### 2.2 Sodium salt

Sodium salts are another important component of organic liquid electrolytes and play a vital role in the performance of electrolytes (Che et al., 2017). For the selection of sodium salt. Firstly, the sodium salt should have sufficient solubility and dissociation ability in the solvent, and the dissociated cations should be free to move without obstacles to provide sufficient charge carriers. Secondly, the sodium salt should remain electrochemically stable within a certain electrochemical window without oxidation or reduction. The sodium salt and the solvent together determine the redox potential of the electrolyte, and the salt anion and the solvent are coupled by electrostatic interaction, which affects the oxidative stability of the electrolyte. In addition, the sodium salt should have good chemical stability as well as safety, remaining chemically inert to the diaphragm, solvent, electrode and collector fluid. If the sodium salt can effectively promote the formation of SEI film at the interface between electrode and electrolyte, it can better enhance the electrochemical performance of electrolyte, such as cycling stability (Strauss, 1993; Borodin and Jow, 2010; Ponrouch et al., 2015; Eshetu et al., 2019; Fadel et al., 2019). The commonly used sodium salts are sodium perchlorate (NaClO_4_), sodium tetrafluoroborate (NaBF_4_), sodium hexafluoroborate (NaPF_6_), sodium trifluoromethanesulfonate (NaCF_3_SO_3_, abbreviated as NaOTf), sodium bis(fluorosulfonyl)imide [Na(FSO_2_)_2_N, abbreviated as NaFSI] and sodium bis(trifluoromethanesulfonyl)imide [Na(CF_3_SO_2_)_2_N, abbreviated as NaTFSI] (Hu et al., 2020). The advantages and disadvantages of these sodium salts are shown in Table 2.

**TABLE 2:** Physical and chemical properties, advantages and disadvantages of commonly used sodium salts (Devlin and Herley, 1987; Ponrouch et al., 2012; Bhide et al., 2014; Evans et al., 2014; Ponrouch et al., 2015; Eshetu et al., 2016; Eshetu et al., 2019; Forsyth et al., 2019; Goktas et al., 2019; Minh Phuong et al., 2019; Hu et al., 2020; Wang et al., 2020).

Name of sodium salt	Anion structure	Molecular weight/·mol^-1^	Melting point/°C	Advantages	Disadvantages
NaClO_4_	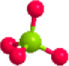	122.4	468	Strong oxidation resistance, suitable for high voltage systems	Explosive in the dry state and difficult to remove the moisture contained
NaBF_4_	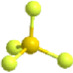	109.8	384	High thermal stability, good safety, easy to make aluminum foil passivation	Harder to dissociate in solvents, low electrical conductivity
NaPF_6_	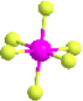	167.9	300	High solubility and high conductivity in different kinds of solvents, easily passivates aluminum foil	Poor chemical stability, easily decomposes to NaF and PF_5_
NaSO_3_CF_3_ (NaOTf)	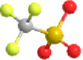	172.1	248	high oxidation resistance and thermal stability	Easy formation of ion pairs in organic solvents, low conductivity of electrolyte
Na[(FSO_2_)_2_N](NaFSI)	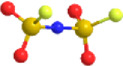	203.3	118	High electrical conductivity, good thermal stability	Narrow electrochemical window, aluminum foil corrosion occurring around 3.8 V
Na[(CF_3_SO_2_)2N] (NaTFSI)	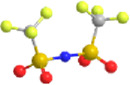	303.1	257	High electrical conductivity, good thermal and water stability, and oxidation resistance	Severe aluminum-collector corrosion

Based on the fact that each of the commonly used sodium salts has its own advantages and disadvantages, which are difficult to overcome. Hybrid systems combining two or more sodium salts have also been investigated, and the aim of avoiding disadvantages is expected to be realized, but the results are not significant. Therefore, it is necessary to develop new sodium salts (Hu et al., 2020). Some new salts that have been synthesized and reported are sodium difluorooxalate borate (NaODFB), sodium 4,5-dicyano-2-(trifluoromethyl)imidazolate (NaTDI), sodium 4,5-dicyano-2-(pentafluoroethyl)imidazolate (NaPDI), sodium bisoxalate borate (NaBOB), sodium bis [salicylato (2-)]-borate (NaBSB), sodium salicylic benzylic acid borate (NaBDSB), sodium tetraphenyl borate (NaBPh_4_), *etc.*

### 2.3 Additives

Additives are the third main component of organic liquid electrolytes. Additives are components that are present in small amounts (less than 5%) in the electrolyte and are characterized by high specificity and small dosage. By adding a small amount of additives, it is possible to make up for the deficiencies of the original electrolyte and significantly optimize the specific performance of the battery without increasing the production cost or changing the production process (Xu, 2014; Wang et al., 2020). According to the function of additives, they are divided into film-forming additives, flame retardant additives, overcharge protection additives and other types of additives (Zhang, 2006).

The most studied are film-forming additives, which are usually easily consumed. During the initial activation cycle they participate and contribute to the formation of the interface between the electrode and the electrolyte, leaving a chemical signal only at the interface and not in the electrolyte itself. The ideal film-forming additives should have higher Fermi energy (E_g_) located in the highest occupied molecular orbital-lowest unoccupied molecular orbital gap than solvents, electrolyte salts, *etc.*, so that they preferentially undergo oxidation or reduction, which in turn improves the film-forming quality and efficiency of the SEI film and effectively enhances the electrochemical performance of the cell (Goodenough and Kim, 2010; Zhu et al., 2017; Niu et al., 2019). The mechanism of action of various additives is shown in Table 3. Other types of additives including acidity enhancers, impurity scavengers, viscosity reducers, free radical scavengers, *etc.*, Also have potential applications in sodium ion batteries (Ponrouch et al., 2015).

**TABLE 3:** Mechanism of action of different types of additives.

Additive type	Name	Mechanism of action	References
Film-forming additives	Vinylidene carbonate (VC)	VC contains unsaturated double bonds that can be broken above the PC and EC decomposition potentials, resulting in macromolecular network polymers that participate in the formation of SEI films	Hwang et al. (2017b)
	Fluoroethylene carbonate (FEC)	The central atom of the FEC has a strong electron-acquisition ability because of the strong electron-absorption effect of the halogen atoms. At high potentials on the anode surface, the electrons of the central atom can be reduced, resulting in a stable SEI film	Komaba et al. (2011a); Darwiche et al. (2012); Qian et al. (2012); Qian et al. (2013); Wang et al. (2014); Yabuuchi et al. (2014); Jang et al. (2015); Sadan et al. (2018)
	Propylene-1,3-sulfolactone (PST)	In PST and DTD, the central S atoms are more electronegative than C atoms, resulting in the preferential formation of stable SEI films containing S compounds on the anode surface. These additives improve the high and low temperature performance of the cells and reduce the continuous increase of the interfacial impedance	Che et al. (2018)
	Vinyl sulfate (DTD)		
	Sodium difluoroxalate borate (NaODFB)	NaODFB can form NaF with small particle size on the electrode surface, which has good film-forming effect	Yan et al. (2019)
Flame retardant additives	Phosphorus containing flame retardant additives including trimethyl phosphate (TMP), triethyl phosphate (TEP), triphenyl phosphate (TPP), tributyl phosphate (TBP), dimethyl methyl phosphate (DMMP), three (2,2,2-trifluoroethyl) phosphite (TFEP) and ethoxy (pentafluoro) cyclotriphosphonitrile (EFPN)*etc.*	When these flame retardant additives are heated, P-containing radicals with flame retardant properties are released, and the phosphorus-containing radicals then capture the hydrogen in the organic radical chain combustion reaction, terminating the chain reaction and making the combustion of organic electrolytes difficult	Hu et al. (2020)
	Fluorinated flame retardant additives including methyl nonafluorobutyl ether (MFE), perfluorinated (2-methyl-3-pentanone) (PFMP) and 1,1,2,2-tetrafluoroethyl-2,2,3,3-tetrafluoropropyl ether (HFE)*etc.*	When encountering an open flame, it is preferred to evaporate and absorb a large amount of heat from the surrounding area, thus extinguishing the flame, and has excellent flame retardancy	Feng et al. (2015b); Zheng et al. (2020)
Overcharge protection additive	Biphenyl (BP)	Prevent overcharging events by accepting additional charge through the redox shuttle	Feng et al. (2015a)

## 3 Current status of research on organic liquid electrolytes

**FIGURE 2:**
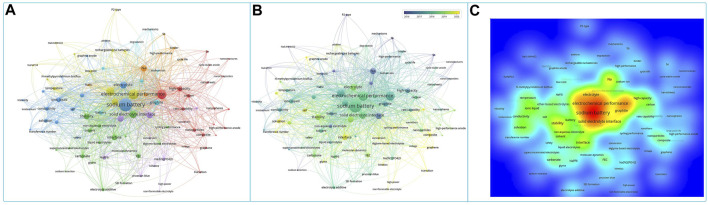
Sodium batteries organic liquid electrolyte keywords co-occurrence analysis: **(A)** network visualization, **(B)** overlay visualization and **(C)** density visualization, produced by vosviewer software.

Sodium ion battery organic liquid electrolytes are classified according to sodium salts and are mainly divided into sodium perchlorate (NaClO_4_)-based organic liquid electrolytes, sodium hexafluorophosphate (NaPF_6_)-based organic liquid electrolytes, sodium bis(fluorosulfonyl)imide (NaFSI)-based organic liquid electrolytes, sodium bis(trifluoromethylsulfonyl)imide (NaTFSI)-based organic liquid electrolytes, and sodium difluoroxalate borate (NaODFB)-based organic liquid electrolytes. Figure 2 shows the keyword clustering analysis of the literature related to organic liquid electrolytes for sodium ion batteries over the years. The overview of research in this field was presented, including electrodes, electrolytes, solid electrolyte interfacial films, and electrochemical properties. In addition to the traditional electrochemical testing of different electrode materials with matching electrolytes, the study of solid electrolyte interfacial membranes and the development of new organic liquid electrolytes are gradually attracting the attention of researchers in recent years. The following section will focus on the compatibility of different types of sodium ion battery electrolytes with each electrode.

### 3.1 Sodium perchlorate (NaClO_4_)-based organic liquid electrolytes

**FIGURE 3:**
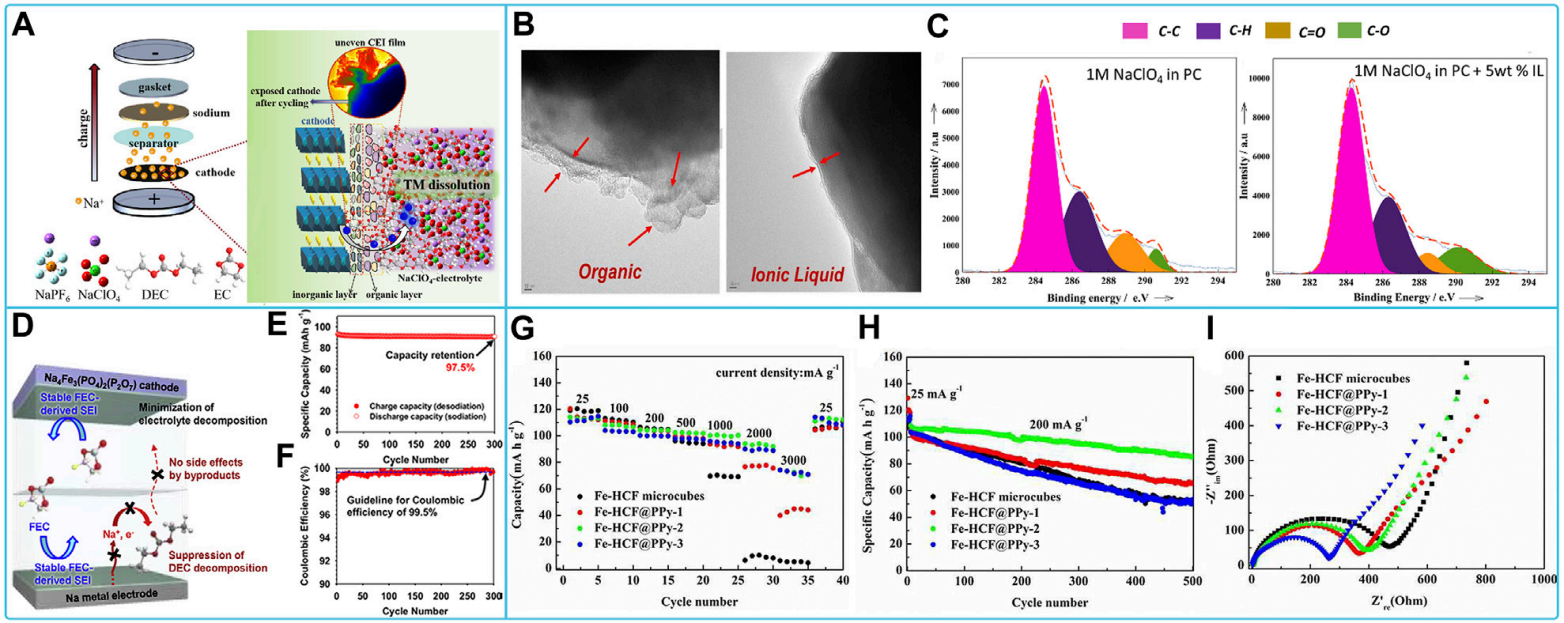
Compatibility of sodium NaClO_4_-based electrolytes with different cathode materials: **(A)** Schematic evolution of CEI film of Na_0.67_Ni_0.15_Fe_0.2_Mn_0.65_O_2_ (N-NFM) cathode in NaClO_4_-based electrolytes (Wang S. et al., 2021). **(B)** HR-TEM images of NVP/C electrodes after 50 cycles with organic electrolyte and organic + IL electrolyte respectively and **(C)** XPS C1s Peaks for NVP/C cathodes after 50cycles with Organic and Organic + IL electrolytes (Manohar et al., 2018a). **(D)** Diagram of the interaction, **(E)** cycling performance and **(F)** Coulombic efficiency of Na_4_Fe_3_(PO_4_)_2_(P_2_O_7_) electrode in NaClO_4_-based electrolyte with FEC (Lee et al., 2016). **(G)** Discharge capacities, **(H)** long-term cycling stability and **(I)** EIS spectra of Fe-HCF and Fe-HCF@PPy electrodes in NaClO4-EC/PC electrolyte (Tang et al., 2016).

NaClO_4_-based organic liquid electrolyte is a widely used electrolyte for sodium ion batteries with good compatibility with common cathode materials (layered oxides, polyanionic compounds, and Prussian blue-like compounds). The battery system consisting of layered oxide and sodium perchlorate-based organic liquid electrolytes exhibited suitable reversible capacity, rate capability, and cycle life. The binary layered oxides P2-Na_x_Co_0.7_Mn_0.3_O_2_ (x ≈ 1) perform well in the electrolyte of NaClO_4_ dissolved in PC with FEC, which is easier to coordinate with the ClO_4_^−^ on the cathode surface than the PC solvent and contributes to the formation of the NaF protective layer on the cathode surface (Cheng et al., 2019). Ternary layered oxides Na_0.67_Ni_0.15_Fe_0.2_Mn_0.65_O_2_ (N-NFM) CEI membranes formed in NaClO_4_ based electrolytes in EC/DEC contain more organic compounds but less inorganic compounds, leading to increased impedance. In addition, CEI membranes are sensitive to perchlorate, which has strong oxidizing properties. A small fraction of the CEI film peels off from the cathode surface, accelerating the dissolution of transition metal (TM) ions and leading to reactivation of electrolyte decomposition (Wang S. et al., 2021). The mechanism of action and subsequent solubilization of TM ions by CEI films is shown in Figure 3A. Therefore, further optimization of electrolyte replenishment is required. Polyanionic compounds have a very solid framework matched to NaClO_4_ electrolytes, allowing for higher cyclability and safety, and have been extensively studied by researchers. For example, Na_3_V_2_(PO_4_)_3_(NVP) showed high discharge capacity and coulomb efficiency with good cycling and rate capability, cycle life in 0.9 M NaClO_4_ dissolved in triethyl phosphate (TEP) electrolyte solution (Liu et al., 2019), and 1 M NaClO_4_ in PC added with 5 wt% [C_3_mpyr] [NTf2] IL (Manohar et al., 2018a). TEP electrolyte has non-flammable and high safety features. The IL additive makes the passivation layer organic and IL-containing and with sulfur in the surface film, and the surface film is more stable (Figures 3B,C). Na_2_VM(PO_4_)_3_ (M = Ga or Al) behaves differently in an electrolyte of 1 M NaClO_4_ with EC/PC = 1:1 v/v, FEC of 5 vol%, which is attributed to the fact that Na_3_VAl(PO_4_)_3_ possesses a larger diffusion bottleneck to transfer more electrons than Na_3_VGa(PO_4_)_3_ and exhibits a higher redox reaction potential (Wang Q. et al., 2021). Therefore, the V^5+^/V^4+^ redox reaction can be induced by the substitution of smaller-sized low-valent (≤+3) cations, which improves the utilization efficiency of the V^5+^/V^4+^ redox reaction. The Na_4_Fe_3_(PO_4_)_2_(P_2_O_7_) cathode formed a protective surface film in the EC/PC/DEC (5/3/2, v/v/v) electrolyte with 0.5 M NaClO_4_ added with FEC and prevented undesirable decomposition of the linear carbonate, leading to excellent cycling performance of the cathode (Figures 3D–F) (Lee et al., 2016). The discharge capacity, impedance, multiplicity performance, cycling stability, and capacity retention of Prussian blue-like cathode materials sodium hexacyanoferric (Fe-HCF) composites coated with polypyrrole (PPy) were greatly improved in the EC/PC electrolyte of NaClO_4_ (Figures 3G–I) (Tang et al., 2016). The insertion of Na^+^ in a series of Prussian blue compounds in organic carbonate electrolytes of NaClO_4_ was investigated. KFe [Fe(CN)_6_] provides the highest reversible capacity at ∼3.6 V for both the high-spin and low-spin Fe^3+^/Fe^2+^ couples (Lu et al., 2012; Wang Q. et al., 2022).

**FIGURE 4:**
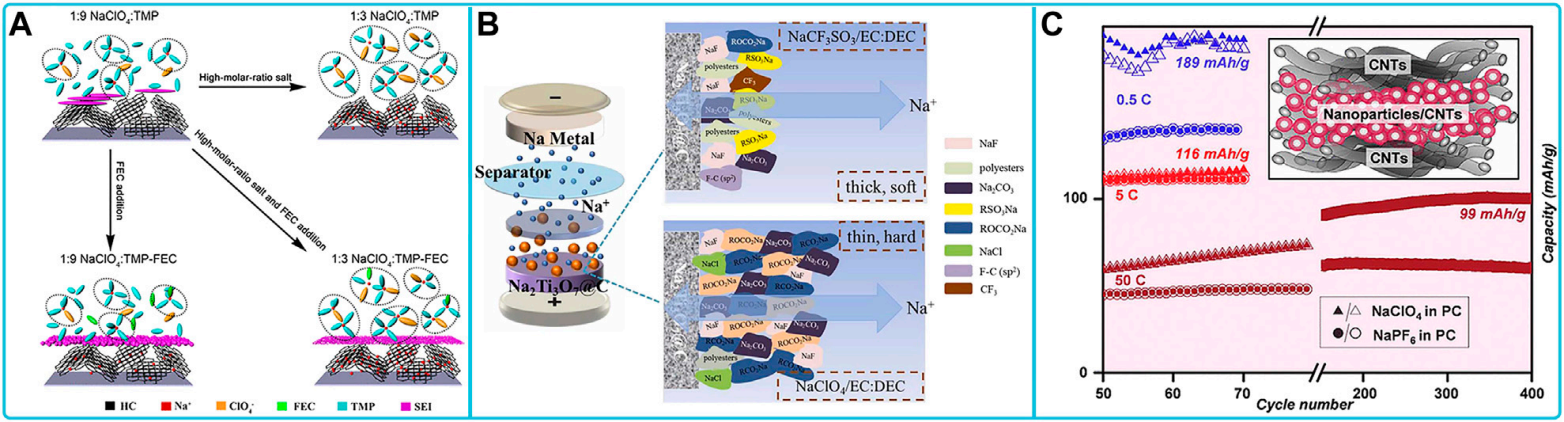
Compatibility of sodium NaClO_4_-based electrolytes with different anode materials: **(A)** Schematic illustration of the behavior of the HC electrode in the different ratios NaClO_4_/TMP electrolytes (Liu X. et al., 2018). **(B)** Schematic evolution of SEI film of Na_2_Ti_3_O_7_@C with different electrolytes (Wen et al., 2023). **(C)** Capacity retention of hollow NP-based γ-Fe_2_O_3_ electrodes at different rates (Koo et al., 2013).

There have also been a number of studies on the compatibility of sodium perchlorate-based organic liquid electrolytes with anode. The most commonly used anode materials are intercalation type materials (e.g., hard carbon). Hard carbon as the anode of sodium ion batteries showed good compatibility with NaClO_4_ electrolytes with solvent of EC, PC, DMC and DEC exhibiting high specific capacity and good capacity retention. The electrochemical properties of hard carbon in electrolyte are further enhanced by the action of some additives (e.g., 1-ethyl-3-methylimidazolium bis(fluoromethanesulfonyl)imide (EMImFSI) (Benchakar et al., 2020), N,N-diethyl-N-methoxyethylammonium bis(trifluoromethanesulfonyl)imide (DEMETFSI) (Egashira et al., 2012), FEC (Liu X. et al., 2018; Pan et al., 2018; Rangom et al., 2019)). For example, The reversibility of sodium insertion became evident at a volume content of 70% of DEMETFSI (Egashira et al., 2012). FEC promotes the formation of the initial sodiuming process SEI and improves the cycle life. By using added FEC and high salt-to-solvent molar ratio TMP electrolytes, it is possible to achieve both low R_SEI_ and very small R_ct_ on HC electrodes, thereby simultaneously inhibiting TMP decomposition and building thin and dense SEI membranes (Figure 4A). The electrolyte with a 5 vol% FEC ratio of 1:3 NaClO_4_/trimethyl phosphate (TMP) exhibited considerable reversible capacity (238 mAh g^-1^ at 20 mA g^-1^), long-term cycle life up to 1,500 cycles, 84% capacity retention at 200 mA g^-1^, and high rate capability (Liu X. et al., 2018). Graphite-based intercalated anode perform well in electrolytes with solvent combinations of EC/DEC (Luo et al., 2015), EC/DMC (Wang S.-W. et al., 2017), and PC (Wang et al., 2013). In addition to carbon materials, other inserted anodes have been investigated, such as TiO_2_, Na_2_Ti_3_O_7_. TiO_2_ in 1M NaClO_4_ in EC/PC electrolyte exhibits the best high-magnification performance of all titanium-based sodium ion anode materials reported so far (Wu et al., 2014). The SEI film generated in Na_2_Ti_3_O_7_@C anode material with sodium perchlorate (NaClO_4_)-based EC/DEC electrolyte contains more Na_2_CO_3_ and NaF, which is caused by the continuous decomposition of NaClO_4_ salt in carbonate solvent. This makes the SEI film thicker and the interfacial impedance greater, leading to a decrease in cell cycling performance, this is in contrast to the NaOTf electrolyte (Figure 4B) (Wen et al., 2023). Metal oxides/sulfides/phosphides are typical conversion electrodes in SIBs, which typically suffer from poor conversion reaction reversibility and shuttle effects. Similar to other electrode materials, stable cycling of conversion electrodes relies on dense SEIs to provide stability and suppress losses of high mechanical strength actives. Hollow γ-Fe_2_O_3_ nanoparticles in PC electrolytes of NaClO_4_ showed superior performance in terms of capacity retention, Coulomb efficiency, multiplicative performance, and cycle life (Figure 4C) (Koo et al., 2013).

In general, NaClO_4_-based organic liquid electrolyte has been widely used as a relatively mature electrolyte for sodium ion batteries. NaClO_4_ electrolyte has contributed to the improvement of energy density of sodium ion batteries with its own stability and oxidation resistance along with some high-voltage cathode materials. However, the compatibility with some positive and negative electrodes is not very good, and it will promote the dissolution of transition metal ions as well as the generation of thick SEI films, which may require a combination of additives and different solvent combinations to optimize the electrolyte in the future.

### 3.2 Sodium hexafluorophosphate (NaPF_6_)-based organic liquid electrolytes

**FIGURE 5:**
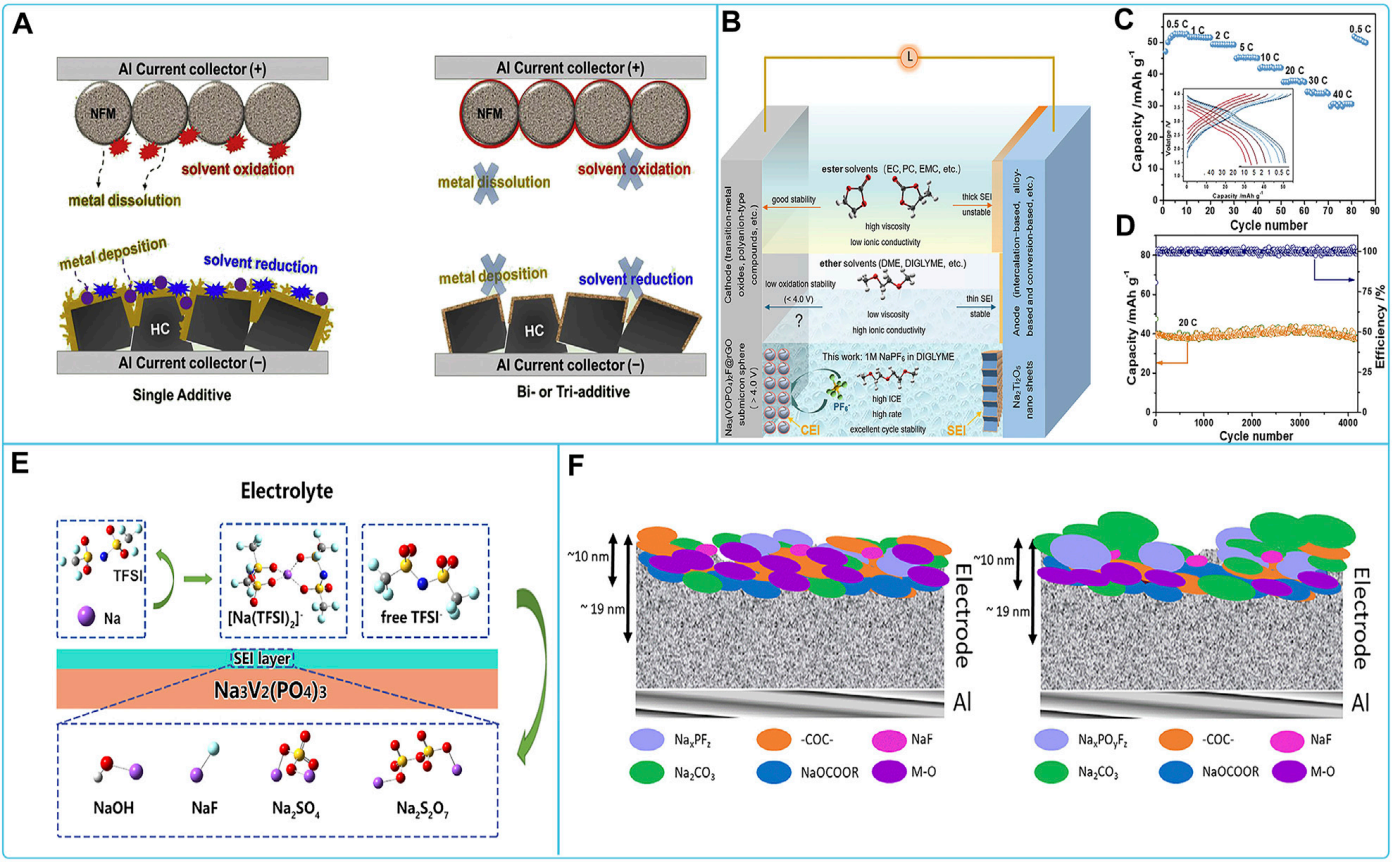
Compatibility of sodium NaPF_6_-based electrolytes with different cathode materials: **(A)** Schematic summary on the role of PST and DTD additives in NFM/HC full cell (Che et al., 2018). **(B)** Illustration of effects of electrolyte system, **(C)** rate performance and **(D)** cycling performance on NVOPF@rGO//Na_2_Ti_2_O_5_ full cell (Ba et al., 2022). **(E)** The diagram of SEI layer formation mechanism on the Na_3_V_2_(PO_4_)_3_ cathode in NaPF_6_/BMITFSI IL electrolyte (Wu et al., 2018). **(F)** Schematic of the CEI on the Na_4_Co_3_(PO_4_)_2_P_2_O_7_ electrode after 1 st Na^+^ extraction and insertion (Zarrabeitia et al., 2021).

NaPF_6_-based organic liquid electrolyte is also one of the commonly used electrolytes for sodium ion batteries. Studies on related cathode materials are often paired with NaPF_6_-based organic liquid electrolytes for a series of electrochemical tests. First is the layered oxide cathode material. The layered oxide exhibits excellent retention and outstanding multiplicative performance, specific capacity, capacity retention and long-term cycling stability in NaPF_6_-based electrolytes. Among them, The CEI film formed by Na_0.67_Ni_0.15_Fe_0.2_Mn_0.65_O_2_ (N-NFM) in NaPF6-based electrolyte is dense and homogeneous, which effectively inhibits the dissolution of transition metal ions and provides a low-energy barrier for Na^+^ transport (Wang S. et al., 2021). The NaNi_1/3_Fe_1/3_Mn_1/3_O_2_ cathode material, paired with a hard carbon anode, maintains up to 92.2% capacity after 1,000 cycles at 1C between 2.0 V and 3.8 V using an optimized electrolyte of 1 M NaPF_6_ dissolved in 1:1 (v/v) PC-EMC +2 wt% FEC, 1 wt% PST, and 1 wt% DTD. The PST and DTD additives promote the formation of a robust SEI on the anode and prevent the dissolution of transition metal ions by inducing the formation of a dense and dense electrolyte (Figure 5A) (Che et al., 2018). The second type of cathode material that is often adapted to NaPF_6_ electrolytes is the polyanionic compound cathode material. Polyanionic compounds matching NaPF_6_ ester and ether electrolytes have been reported. Na_3_(VOPO_4_)_2_F (NVOPF)/rGO 3D sub-microspheres and Na_2_Ti_2_O_5_ nanosheet anode electrode and NaPF_6_ diglyme electrolyte, the full cell was further designed with high initial Coulombic efficiency (90%), excellent multiplicative performance (40°C) and ultra-stable cycling performance (>4,000 cycles without degradation) (Figures 5C,D). The electrolyte defines a robust fluorine-rich inorganic-organic interface, which effectively improves the interface and promotes ultra-fast charge transfer (Figure 5B) (Ba et al., 2022). The SEI layer between Na_3_V_2_(PO_4_)_3_ and NaPF_6_/1-butyl-3-methylimidazolium bis (trifluoromethanesulfonyl) imide (BMITFSI) ionic liquid electrolyte consists of NaOH, Na_2_SO_4_, Na_2_S_2_O_7_ and NaF (Figure 5E). This is the reason for its good electrochemical properties (Wu et al., 2018). Half-cell tests of Na_4_Co_3_(PO_4_)_2_P_2_O_7_ in an electrolyte solution of EC/DEC with 1 M NaPF_6_ showed the formation of a double layer in the fully Na^+^ extracted state of charge, with semi-organic-rich material found in the subsurface region near the electrode and more organic material in the outermost surface region near the electrolyte. At the same time, an additional outermost inorganic cover layer consisting of sodium carbonate and sodium fluorophosphate was formed after complete Na^+^ insertion (Figure 5F). Therefore, the Na_4_Co_3_(PO_4_)_2_P_2_O_7_ cathode provided excellent cycling performance (Zarrabeitia et al., 2021). The third cathode material used to match the NaPF_6_-based electrolyte is a Prussian blue-like compound. The Prussian blue cathode electrode exhibits enhanced capacity retention in a volume ratio of 7: 3 of di-(2,2,2 trifluoroethyl) carbonate (TFEC)/fluoroethylene carbonate (FEC) consisting of 0.9 mol L^-1^ NaPF_6_. The electrolyte has excellent flame retardancy and good compatibility with sodium electrodes. The polycarbonate formed on the cathode surface plays an important role in the studied electrolyte system by enhancing the ionic conductivity and reducing the impedance of the solid electrolyte interphase (SEI) layer (Zeng et al., 2020).

**FIGURE 6:**
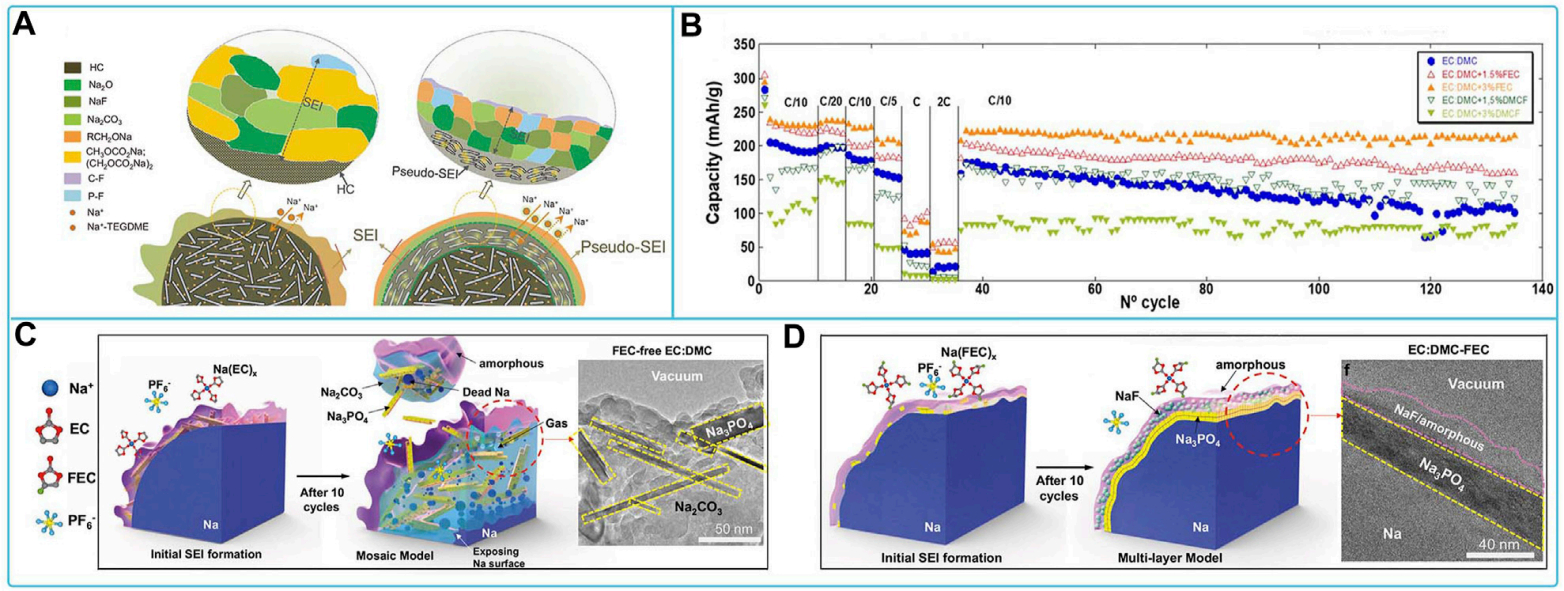
Compatibility of sodium NaPF6-based electrolytes with different anode materials: **(A)** Schematic illustration of SEI and pseudo-SEI structures and chemistry for Na^+^ storage in HC anodes in NaPF_6_ based ester and ether electrolytes (Ma et al., 2021). **(B)** Cycle capacity for HC electrodes using 1 M NaPF6 in EC:DMC with addition of 1.5% and 3% of FEC or DMCF (Fondard et al., 2020). **(C, D)** Representation of free FEC and FEC electrolyte additives in tuning the microstructure of SEI on Na metals during cycling, respectively (Han et al., 2021).

Researchers often choose NaPF_6_-based organic liquid electrolytes for the study of anode materials (intercalation-type, conversion-type materials, and non-metallic materials). Hard carbon HC performs well in NaPF_6_ electrolytes with ester and ether solvents. Among them, The TEGDME-based NaPF_6_ electrolyte can exhibit excellent rate capability, capacity retention and cyclic Coulombic efficiency at the HC anode. This is because the stable layer-by-layer SEI in the TEGDME-based electrolyte combined with the solvent layer “pseudo-SEI” on the HC facilitates high-performance Na^+^ ion storage in the HC and extends the cycle life of the HC anode material (Figure 6A) (Ma et al., 2021). In the EC/DMC electrolyte, the use of 3% FEC significantly increased the total capacity and capacity retention, while the use of DMCF additive had a negative impact on capacity but provided better cycling performance than the additive-free electrolyte (Figure 6B) (Fondard et al., 2020). The good performance can be attributed to the SEI composition consisting mainly of sodium ethylene dicarbonate NaO_2_CO-C_2_H_4_-OCO_2_Na (NEDC) and NaF. FEC as an additive promotes the production of NaF, which enhances the NEDC-rich SEI and results in a significant increase in capacity retention during cycling. Graphite-based materials have good reversible capacity and good cycling performance in NaPF_6_/diethylene glycol dimethyl ether (DEGDME) electrolytes (Han et al., 2015; Cabello et al., 2017; Nacimiento et al., 2019; Li Z. et al., 2021; Zheng et al., 2022). Transformation-based electrode materials (e.g., copper phosphorothioate (Cu_3_PS_4_) (Brehm et al., 2020), dandelion-shaped manganese sulfide (DS-MnS) (Duong Tung et al., 2018), tin phosphide (Sn_4_P_3_/C) electrodes (Luis Gomez-Camer et al., 2019), TiS_2_ (Tao et al., 2018), *etc.*) are also often studied matching NaPF_6_-based organic liquid electrolytes. The choice of solvent is often ether-based solvents (diethylene glycol dimethyl ether, DME), and to a lesser extent, esters. In these electrolytes the converted electrode materials exhibit high capacity, cycling performance, multiplicative performance, first turn Coulomb efficiency, *etc.* It indicates that the conversion-type electrodes have high compatibility with ether-based NaPF_6_ electrolytes. Non-metallic elemental anode materials [Micron Pb particles (Darwiche et al., 2016), and Bi electrodes (Wang C. et al., 2017; Li Y. et al., 2021)] exhibit good electrochemical performance in NaPF_6_-based diethylene glycol dimethyl ether electrolytes and good compatibility with NVP and NVPF cathode materials, and the full-cell test solution exhibited good performance. For the study of sodium metal electrodes in NaPF_6_-based electrolytes, EC/DMC is often chosen as the solvent. When FEC is added, the Na metal electrode forms a multilayer SEI structure, including an external NaF-rich amorphous phase and an internal Na_3_PO_4_ phase. This layered structure stabilizes the SEI and prevents further reactions between the electrolyte and the Na metal. Without FEC, the carbonate-based electrolyte containing NaPF_6_ reacts with the metal electrode to produce an unstable SEI, rich in Na_2_CO_3_ and Na_3_PO_4_, which continuously depletes the cell’s sodium reserves during cycling (Figures 6C,D) (Han et al., 2021). The Na metal deposition/dissolution efficiency increased with increasing NaDFP concentration when sodium difluorophosphate NaDFP additive was added. NaDFP suppressed the overpotential and interfacial resistance. A high multiplicative capacity and long cycle life of 76.3% capacity retention after 500 cycles were achieved with 1 wt% NaDFP. The NaDFP-containing electrolyte formed a more stable SEI layer than the pure electrolyte, thus mitigating further degradation of the electrolyte (Yang et al., 2021). In specific glyme (chain ether) electrolytes, the sodium-metal interface produces a thin, homogeneous inorganic SEI composed primarily of Na_2_O and NaF that may not support extensive or extreme cycling conditions, but the addition of FEC provides a more robust SEI to facilitate a large number of consistent sodium plating and stripping cycles (Seh et al., 2015; Sarkar et al., 2023).

In general, NaPF_6_-based organic electrolytes, as a commercially available electrolyte, have good compatibility with common cathode and anode materials. NaPF_6_ shows superior performance in ether solvents compared to other sodium salts. However, the study of NaPF_6_-based electrolyte body solutions (e.g., solvation structure, sodium ion transport kinetics) is still at the beginning stage and will be further enhanced to reveal the reasons for their superior electrochemical performance in the future.

### 3.3 Sodium bis(fluorosulfonyl)imide (NaFSI) based organic liquid electrolyte

**FIGURE 7:**
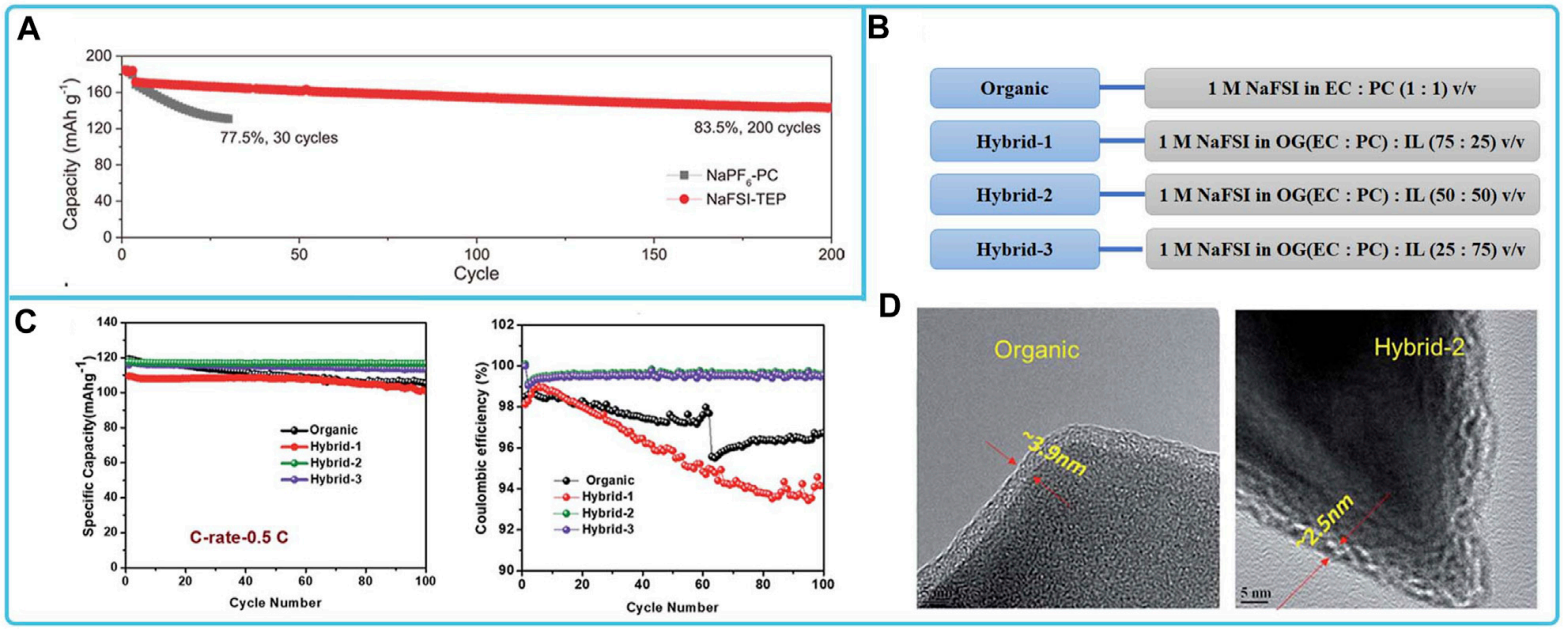
Compatibility of sodium NaFSI-based electrolytes with different cathode materials: **(A)** Long-cycling performance of hard-carbon||NaNMC full cells in two electrolytes (Jin et al., 2022). **(B)** Organic and hybrid electrolyte compositions, **(C)** cycling performance and Coulombic efficiency of NVP@C with organic and hybrid electrolytes at 0.5C and **(D)** HR-TEM images of NVP@C electrodes after cycling with the organic and hybrid-2 electrolyte, respectively (Manohar et al., 2018b).

The cathode materials matched to NaFSI electrolytes mainly include layered oxides, polyanionic compounds, and organics. For the compatibility study of layered oxide cathode materials with NaFSI-based organic liquid electrolytes, the solvents used with NaFSI are esters, ethers, ionic liquids. Among them, NaNi_0.68_Mn_0.22_Co_0.10_O_2_ (NaNMC) exhibited good long-cycle performance and capacity retention in the phosphate electrolyte NaFSI-TEP (Figure 7A). The stable cycling can be attributed to the formation of a stable CEI layer on the NaNMC cathode, which suppresses the surface reconstruction of the cathode, the dissolution of transition metals at the cathode, and the persistent side reactions at the electrolyte/electrode interface (Jin et al., 2022). NaFe_0.4_Ni_0.3_Ti_0.3_O_2_ matches well in electrolytes using IL solvents (e.g., C_3_C_1_pyrrFSI). This ionic liquid electrolyte enhances the formation of passivation layer on the surface of Al current collectors, stabilizes the surface to 5 V, and prevents Al corrosion even at 55°C (Otaegui et al., 2015). For the compatibility study of polyanionic compounds with NaFSI electrolytes, NVP in NaFSI electrolytes with ester solvents and ionic liquids has high Coulombic efficiency, fast charging capability, stable cycling, high reversible capacity and capacity retention (Jian et al., 2013; Manohar et al., 2018b; Zheng et al., 2018; Li et al., 2022). Among them, the ionic liquid can promote the formation of a stable and thin SEI layer on the surface, which improves the discharge capacity and cycling performance of NVP@C cathode materials (Figures 7C,D) (Manohar et al., 2018b).

**FIGURE 8:**
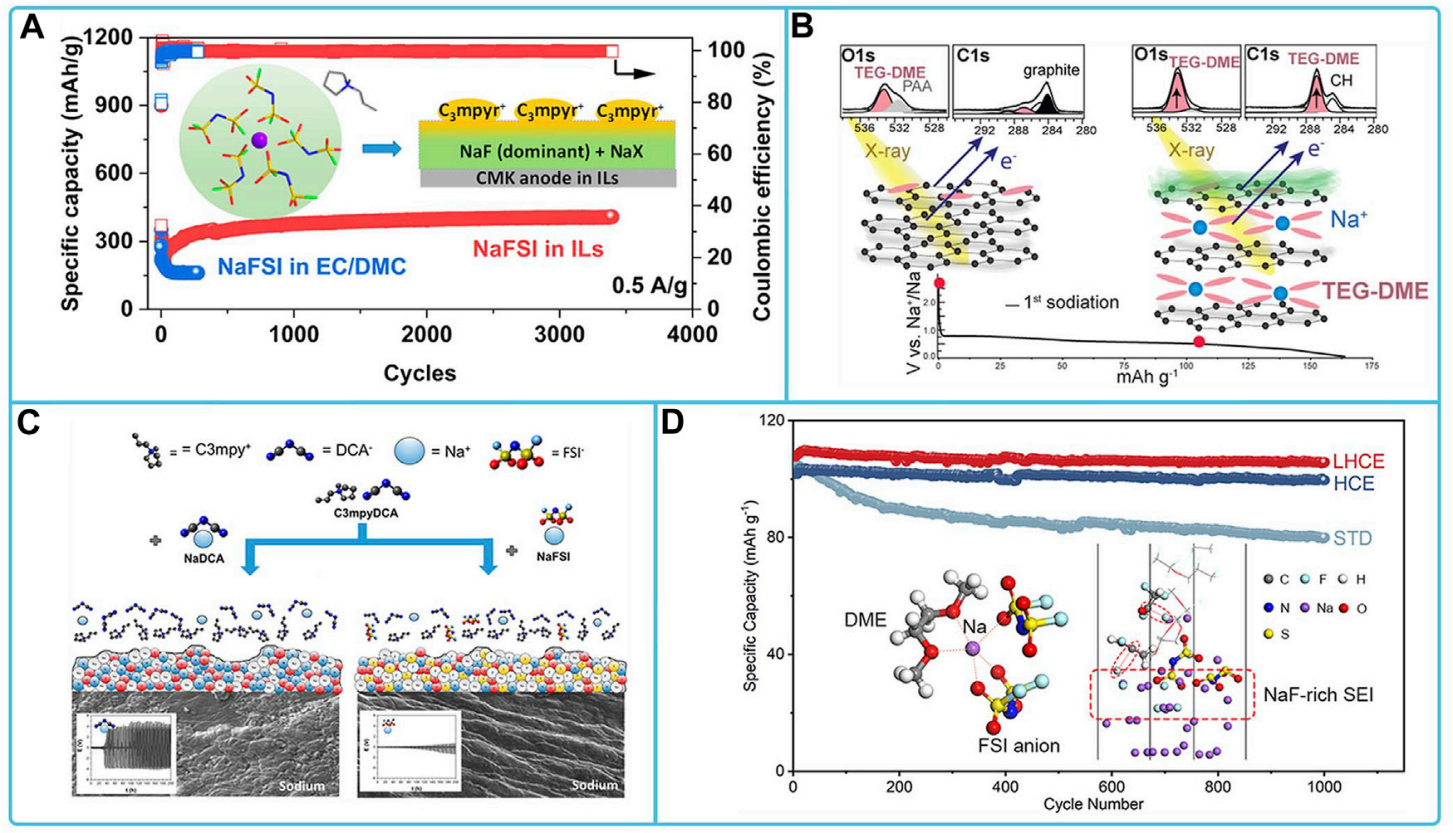
Compatibility of sodium NaFSI-based electrolytes with different anode materials: **(A)** cycling stability tests of Na/CMK cells in carbonate and ionic liquid electrolytes (Sun et al., 2021). **(B)** Schematic representation of the surface processes occurring at this particular graphite/TEG-DME electrolyte interface (Maibach et al., 2017). **(C)** Schematic representation of the sodium-solvated and interfacial structures of NaFSI and NaDCA on sodium-metal surfaces in the [C_3_mpyr]DCA IL systems (Forsyth et al., 2019). **(D)** Cycling performance Na/NVP in NaFSI/DME electrolyte with TTE using STD, HCE, and LHCE, and schematic of Na^+^ solventization and formation of SEI on sodium metal surface (Wang Y. et al., 2021).

The anode materials matched to NaFSI electrolyte mainly include intercalation type materials, alloy type materials, conversion type materials and Na metal electrodes. The NaFSI electrolytes suitable for the study of intercalation-type materials include ester electrolytes, ether electrolytes, and ionic liquid electrolytes. Among them, in 3 mol dm^-3^ NaFSI/PC + EC electrolyte, an organic-inorganic equilibrium SEI (mainly composed of (CH_2_)_n_ and NaF) was formed on the surface of the hard carbon electrode. This SEI not only enables easy charge transfer and fast Na^+^ transport, but also exhibits strong passivation ability and excellent durability (Patra et al., 2019). CMK half-cells exhibit extraordinary cycling stability and high reversible capacity in a 3.8 M NaFSI IL electrolyte in C_3_mpyrFSI (Figure 8A). This is due to the contribution of anionic decomposition species in ILs leading to inorganic SEI on mesoporous carbon CMK electrodes with high ionic conductivity, which promotes Na^+^ desolvation and diffusion kinetics. This rapid Na + migration facilitates improved reaction rates and cycling stability (Sun et al., 2021). Graphite materials do not perform well in the ether electrolyte of NaFSI, where side reactions occur between the electrolyte and the graphite electrode, and the formation of SEI films, which consist mainly of salt decomposition products and hydrocarbons, as shown in Figure 8B, leading to a low Coulomb efficiency of the studied cell system (Maibach et al., 2017; Goktas et al., 2019). For the study of Na metal electrodes in NaFSI-based organic electrolytes, electrolytes that have been reported are high concentration electrolytes with NaFSI and ester and ether electrolytes with additives. Among them, The Na|| Na_3_V_2_(PO_4_)_3_ cell is stable for nearly 1,400 cycles at 2 C in a highly concentrated electrolyte of DME with the addition of a small amount of SbF_3_ at 4 mol L^-1^ NaFSI and also exhibits excellent multiplicative performance of 80 mAh g^-1^ at 40°C. This is because the SbF_3_ additive forms a hard Na-Sb alloy layer, while the high concentration contributes to the formation of a dense NaF-rich SEI layer on the Na metal surface. This bilayer structure of the SEI layer effectively prevents dendrite growth and provides fast interfacial ion transport (Fang et al., 2020). The addition of NaFSI to methyl propylpyrrole dicyandiamide ([C_3_mpyr]DCA) ionic liquid produces a more stable SEI layer (Figure 8C) (Forsyth et al., 2019). The addition of 1,1,2,2-tetrafluoroethyl-2,2,3,3-tetrafluoropropylether (TTE) to 3.8 M NaFSI/DME electrolyte forms a localized high concentration electrolyte (LHCE), which helps to construct a stable SEI for SMBs. TTE also decomposes on Na metal anodes, synergistically forming dense SEI with low surface resistance and good mechanical properties, rich in NaF, which facilitates the transport of Na^+^ ions and inhibits the growth of Na dendrites (Figure 8D) (Wang Y. et al., 2021). Transformation-based anode materials (e.g., Cu_1.8_S/C (Li H. et al., 2021), SnP nanocrystals (NCs) (Liu J. et al., 2018), tin phosphide (Sn_4_P_3_) (Mogensen et al., 2017; Mogensen et al., 2018)) in FSI-based organic electrolytes exhibited stable cycling ability and high capacity. Both NaFSI and FEC additives contribute to the formation of a stable NaF-rich SEI on the anode surface.

In general, NaFSI electrolytes dissolved in carbonate ester and ionic liquids exhibit better electrochemical performance and have better compatibility for hard carbon electrodes and sodium metal electrodes compared to other sodium salt electrolytes. In the future, combining different types of cathode and anode electrodes to assemble a complete battery for electrochemical testing, as well as contributing to the design and implementation of flame-retardant batteries, may be the trend for this electrolyte.

### 3.4 Sodium bis(trifluoromethylsulfonyl)imide (NaTFSI)-based organic liquid electrolyte

The cathode materials adapted to NaTFSI-based organic liquid electrolytes for electrochemical testing mainly include layered oxides and polyanionic compounds. Layered oxides (P2-type Na_2/3_Ni_1/3_Mn_2/3_O_2_ (Risthaus et al., 2018), Na_0.45_Ni_0.22_Co_0.11_Mn_0.66_O_2_ (Chagas et al., 2014), and Na_0.44_MnO_2_ (Stigliano et al., 2022)) exhibited high discharge capacity and capacity retention in the ionic liquid electrolyte with NaTFSI. For the polyanionic compound, NaFePO_4_/Na half-cells in sodium bis(trifluoromethanesulfonyl)imide (NaTFSI)-bonded butylmethylpyrrolidine (BMP)-TFSI ionic liquid (IL) electrolyte operate at 3 V. This IL electrolyte shows high thermal stability and non-flammability. NaFePO_4_ has the best capacity at 50 °C in 0.5 M NaTFSI mixed IL electrolyte (Wongittharom et al., 2014).

**FIGURE 9:**
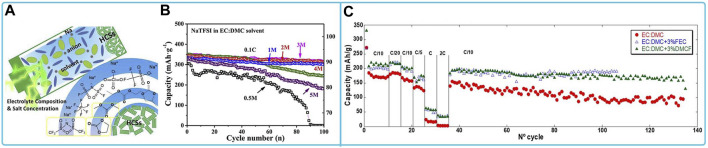
Compatibility of sodium NaFSI-based electrolytes with HCSs materials: **(A)** Schematic diagram of the interaction mechanism of HCSs electrodes in ultra-high concentration electrolytes and **(B)** cycling performance at 0.1C of HCSs in electrolytes with different salt concentrations (Chen et al., 2020). **(C)** Cycling capacity for HC electrodes using 1 M NaPF_6_ in EC:DMC with addition of 1.5% and 3% of FEC or DMCF(Fondard et al., 2020).

The most commonly used anode material for NaTFSI organic electrolyte is hard carbon. NaTFSI organic electrolytes used for hard carbon electrodes include ester electrolytes and ionic liquid electrolytes. Among them, hard carbon electrodes in 2 M NaTFSI/EC:DMC electrolyte provided the best initial reversible capacity, high electrochemical stability, and good cycling stability. In addition, a sharp capacity decay was observed after cycling in an ultra-high concentration electrolyte (5 M NaTFSI/EC: DMC) (Figures 9A,B) (Chen et al., 2020). In NaTFSI/EC: DMC electrolyte, the addition of FEC or DMCF was found to be beneficial for overall capacity and capacity retention during cycling (Figure 9C) (Fondard et al., 2020).

In addition, some applications of NaTFSI-based electrolytes in full batteries have been reported. For example, the full cell consisting of Na_3_V_2_(PO_4_)_2_F_3_ cathode and (Na_2+x_Ti_4_O_9_/C) anode exhibited high capacity retention in a nonflammable low eutectic solvent (DES) including sodium bis(trifluoromethane) sulfonate (NaTFSI) dissolved in N-methylacetamide (NMA). The improved electrochemical stability was associated with a stronger surface film formed at the electrode/electrolyte interface (De Sloovere et al., 2022). P2-Na_0.6_Ni_0.22_Fe_0.11_Mn_0.66_O_2_ cathode and Nanostructured Sb-C composite anode cells in a 0.2M NaTFSIPyr_14_TFSI ionic liquid-based electrolyte exhibited high specific capacity for the full cell. The electrolyte has a high ionic conductivity and high thermal stability. The anodic stability of this electrolyte was up to 4.7 V vs. Na^+^/Na (Hasa et al., 2016).

In general, NaTFSI ionic liquid electrolytes show good electrochemical performance. However, there are some problems, such as corrosion of the aluminum foil, capacity decay and some disadvantages of the ionic liquid electrolyte itself (e.g., high viscosity, poor wettability to the electrode, *etc.*). Efforts are still needed to improve these shortcomings in the future.

### 3.5 Sodium difluoroxalate borate (NaODFB)-based organic liquid electrolyte

**FIGURE 10:**
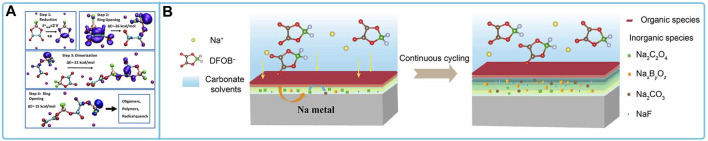
Compatibility of sodium NaODFB-based electrolytes with different electrode materials: **(A)** Pathway of CEI film formation by FeF_3_ in NaODFB-based electrolyte (Chen et al., 2015). **(B)** Schematic illustrations of the NaDFOB-derived SEI structure on the surface of sodium metal (Gao et al., 2022).

Sodium difluoro (oxalato)borate (NaODFB) is a new chelated sodium salt discovered by researchers in recent years. Only a few articles have been published to study this electrolyte. Chen et al. (Chen et al., 2015) found that Na/Na_0.44_MnO_2_ half-cells combined with NaDFOB-based electrolytes exhibited greatly enhanced multiplicative capacity and cycling performance. Sun et al. (Sun et al., 2020) developed a high-capacity nanoconstrained FeF_3_ SIB-based cathode and found that the best cycling performance was achieved using NaDFOB salts in a ternary electrolyte (EC:DEC:DMC), with much higher cycling performance compared to the conventionally used NaClO_4_, which was associated with the formation of a thin and conformal CEI protective film on the cathode. They further predicted that the DFOB anion reduction-mediated radical oligomer/polymer pathway may be an important part of the formation of CEI films. As shown in Figure 10A. Wang et al. (2023) investigated the compatibility of NaODFB electrolyte with NVP cathode.1 M NaODFB-DME electrolyte contributed to the formation of thinner CEI film on the surface of NVP material with low content of B_2_O_3_, which resulted in high specific capacity and capacity retention of the cell. Zhao et al. (2022) investigated the compatibility of NaODFB ether electrolyte with HC anode at high temperature. The Na/HC half-cell with 1 M NaODFB in DME has a high reversible capacity of 249.9 mAh/g at 100 mA/g and 55°C, exhibiting excellent cycling stability attributed to the SEI membrane groups B-F and B-O containing inorganic substances. Gao et al. (2022) found that there was a dense and smooth SEI film on the surface of sodium sheets after cycling in NaODFB-based carbonate electrolyte, and the SEI film could effectively inhibit the growth of sodium dendrites. They provide insight into the underlying mechanism of the protective effect provided by SEI derived from sodium difluoro (oxalate)borate (NaDFOB) (Figure 10B). The pre-reduction of the DFOB^−^ contributes to the formation of SEI and inhibits the decomposition of the carbonate solvent, and the DFOB^−^ is gradually transformed into a borate- and fluoride-rich SEI with cycling. The protective effect of SEI is optimized at 50 cycles, resulting in a threefold increase in the lifetime of the sodium metal batteries.

In summary, NaDFOB has high compatibility with various common solvents used for NIBs, which means that NaDFOB may be very effective for various electrode materials for other NIBs. However, the preliminary work mainly focused on its application as an additive. We should optimize the electrolyte of NaODFB as the main salt and apply it to the full battery. Further studies on the complex interactions of NaDFOB electrolytes with different solvents with various electrode materials are also necessary. The goal of exploring its full potential as a emerging and high-performance electrolyte for sodium ion batteries will be realized in the future.

## 4 Composition, formation mechanism and regulation strategy of the interface between electrolyte and electrode

The study of the solid-liquid interface formed between electrolyte and electrode material is a hot research topic in the field of batteries. The solid-liquid interface film is formed between the electrolyte and electrode material during the first cycle of charging and discharging, and the presence of the interface film prevents the electrolyte from continuously contacting the electrode material and decomposing, thus allowing the electrochemical window of the electrolyte to be extended. Factors such as the denseness, thickness and components of the solid-liquid interfacial film have a great influence on the cycling performance of the battery, and obtaining a stable interfacial film with protective effect and stable Na^+^ transport has been the goal pursued by researchers.

### 4.1 Composition

In 1979, Peled (1979) found that alkali and alkaline earth metals in non-aqueous batteries form a surface film in contact with the electrolyte, which is an intermediate phase between the metal and the electrolyte and has the characteristics of an electrolyte, and hence the concept of “solid electrolyte interphase (SEI)” was introduced. At this time he considers the SEI model to be a simple two-dimensional passivation film structure. In 1997, Peled et al. (1997) suggested the mosaic model by arguing that insolubles generated from all types of reduction reactions of the electrolyte occurring simultaneously are deposited randomly mixed on the anode and stacked on each other to form a mosaic-like structure. In this model, grain boundaries and interfaces in SEI may act as electron conduction paths to promote the growth of dendrites and electron leakage. In 1999, Aurbach et al. (1999) proposed a multilayer structure of SEI films in lithium-ion battery systems using various means such as infrared spectroscopy, Raman spectroscopy, X-ray photoelectron spectroscopy, and electrochemical impedance spectroscopy, arguing that the passivation film formed at the beginning of the metallic Li surface is unstable and changes during the electrochemical process, with various types of substances forming one by one, and that traces of water in the electrolyte, solute Anion decomposition products also continue to influence the generated SEI film to form a multilayer film structure. This dynamic concept has also been applied to sodium ion batteries to derive bilayer and even multilayer structure models.

It is generally believed that the electrode-electrolyte interfacial film consists of the inorganic layer located on the inside connected to the electrode material and the organic layer located on the outside extending into the electrolyte. The inorganic layer is mostly inorganic with some sodium, and the organic layer is mostly organic with sodium formed by the reaction of solvent molecules with sodium. The formation of such a bilayer structure can be divided into two stages, namely, the formation of a bilayer on the surface when electrons flow into the anode and the participation of electrons in the reaction process. When the anode is filled with electrons, Na^+^ will be enriched on the electrode surface to form a bilayer. At the beginning the passivation film is thin and electron transfer is easy, so the double electron reaction occurs preferentially. The solvent molecules of the solubilized coordinated Na^+^ get electrons to be reduced and are more likely to produce inorganic products such as Na_2_CO_3_ and Na_2_O, which are precipitated on the electrode surface, while at the same time, sodium salt anions or additives may also participate in the reaction to produce NaF, NaCl, NaS, and Na_2_SO_4_, etc (Hu et al., 2020). The positive effect of NaF on dense SEI formation and unstable interphase growth control. Content increases appropriately to suppress the solubility of organic sodium carbonate (NaO_2_CO-C_2_H_4_-OCO_2_Na) and promote the conductivity of Na^+^ through the SEI layer, thus improving the electrochemical properties, whereas an increase in Na_2_CO_3_ content does not (Fondard et al., 2020). F-S or S=O species were also detected in the case of FSI^−^ or bis(trifluoromethane)sulfonylimine (TFSI^−^) anions (Ding et al., 2019). However, as the thickness of the membrane increases, electron transfer is blocked and single-electron reactions begin to dominate, with organic species such as ROCO_2_Na (R is an organic group) organically accumulating on the inorganic layer to form an organic layer. The specific species of the organic and inorganic components depend on the reaction between the electrode surface and the electrolyte. Different electrode materials have different SEI components in different electrolyte systems. The thickness of the SEI film is usually between a few nanometers and tens of nanometers, which is mainly related to the electron tunneling distance, and if there is no surface damage or decomposition, after reaching the longest distance of electron tunneling, the solvent will not be able to continue to get electrons to be reduced and thus stop decomposing, and the thickness change of the SEI film will decrease and become an electron insulator and ion conductor, and stabilized (Hu et al., 2020). Recently, Cui et al. (Zhang et al., 2022) revealed the original structure and redefined the composition of SEI by using advanced cryo-electron microscopy to characterize the swelling state of SEI in various electrolytes, and showed that the swelling behavior depends on the electrolyte type and profoundly affects the ion transport in SEI. In the inorganic-rich SEI, the swelling rate is lower, resulting in a more stable electrochemical cycle of the cell.

The ideal SEI film should have the following characteristics: (i) good electronic insulator, preventing the electrolyte from being oxidized or reduced by charge transfer on the surface; (ii) good sodium ion conductivity, selectively allowing the passage of Na^+^ and preventing the solvent from entering the electrode material or directly contacting the electrode; (iii) good chemical and electrochemical stability, with no side reactions in the cell system; (iv) good thermal stability, stably adhering to the surface of the electrode material even at high temperatures; (v) homogeneous, dense and thin, possessing good mechanical properties and not easily flaking and dissolving (Hu et al., 2020).

### 4.2 Formation mechanism

**FIGURE 11:**
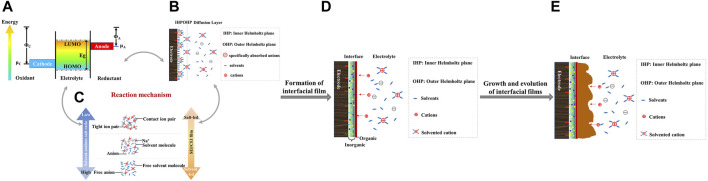
Schematic diagram of **(A)** the energy of the electrode and electrolyte (Gebresilassie Eshetu et al., 2018), **(B)** specific adsorption (Zhang and Zhao, 2009), **(C)** ion solventization (Li et al., 2020), **(D)** interface film formation, and **(E)** interfacial film growth and evolution.

The formation of the interfacial film is mainly the result of a combination of three factors: the energy polarization difference between the electrode and the electrolyte, the specific adsorption behavior and the ion solvation behavior, and the formation is accompanied by the interfacial growth and evolution. The specific formation mechanism is shown in Figure 11. The details of each part are developed below.

The interfacial film arises from the difference in energy states between the two main parts of the electrode and the electrolyte (Gauthier et al., 2015). If the electrochemical potential μ_A_ of the anode is higher than the lowest unoccupied molecular orbital LUMO of the electrolyte, electrons are spontaneously transferred from the anode to the electrolyte, which leads to the reduction of the electrolyte and the formation of the interfacial film. Similarly, when the electrochemical potential μ_C_ of the cathode is lower than the highest occupied molecular orbital level HOMO of the electrolyte, electrons are transferred from the electrolyte to the anode and the solvent molecules of the electrolyte lose electrons leading to oxidation of the solvent, while the anode gains electrons and the transition metal cations (such as Mn^4+^, Ni^4+^, Co^4+^, *etc.*) in the material are reduced (Wang et al., 2020). The interfacial film on the surface of the anode, called SEI, is distinguished from the anode, and the products of electrolyte oxidation decomposition remain on the surface of the cathode, defined as the CEI passivation layer. To ensure a higher energy density, one chooses to initiate the redox reaction of the electrode at a voltage that exceeds the stability of the electrolyte. It is well known that non-aqueous electrolytes typically used in Li-ion batteries are oxidized if the operating voltage is higher than about 4.5 V with respect to Li^+^/Li. Given that the equilibrium potential of Na^+^/Na is 0.3 V higher than that of Li^+^/Li, some commonly used carbonate electrolytes will become thermodynamically unstable if the voltage in SIBs is higher than about 4.2 V (vs. Na^+^/Na) (You and Manthiram, 2018).

Specific adsorption behavior and ion solvation behavior are also the main factors affecting the formation of interfacial layers. The occurrence of specific adsorption behavior precedes the ion solvation behavior, i.e., the strong interaction of some substances with the electrode surface promotes the formation of interfacial films. The interfacial film model includes the inner Helmholtz plane (IHP) and the outer Helmholtz plane (OHP). In general, the specific absorption behavior of unsolvated molecules is mainly present in IHP, while ionic solvated structures are mainly present in OHP (Zhang and Zhao, 2009). The enrichment of specific substances initially adsorbed on the electrode surface determines the initial interfacial composition and structure, while the ion solvation structure subsequently acts to promote the growth of the interfacial layer (Yan et al., 2020). For ionic solventization behavior, the solventized structure is mainly related to the coordination of alkali metal cations (e.g., Na^+^) to electronegative atoms of the solvent molecule (e.g., carbonyl/ether oxygen) or anions (e.g., fluorine in NaPF_6_ salts). Their binding energy depends strongly on the type of cation, anion and solvent. Differences in the solvation structure and diffusion kinetics in different electrolytes subsequently lead to differences in the electrochemical properties of their organic and inorganic interfacial products. In addition, differences in ion solvation structures can lead to different degrees of changes in the electrolyte LUMO energy levels. The solventized Na^+^ structure at the molecular level in the electrolyte can change the preferential decomposition order of the solvent and the anion. As the salt concentration increases, the anions become more involved in the solventized shell layer, driving the transfer of LUMO from the solvent to the anion and forming an inorganic-rich interface (Xing et al., 2018). Thus, changes in the solventization environment will alter the previous order of solvent molecule or anion consumption, determining the initial composition formation of the internal interfacial layer, further affecting the organic/inorganic composition arrangement, structural evolution and overall ion transport capacity. The ionic solventization behavior is the main induction of surface interfacial phase formation and depends on the reduction/oxidation order of solvent molecules or anions (Li et al., 2020). The solvent-induced interfacial layer is dominated by the predominant organic matter in dilute electrolytes. However, the anion-induced interfacial layer in highly concentrated electrolytes consists of more inorganic species, such as NaF, NaCl and Na_2_CO_3_ (Zeng et al., 2018; Zheng et al., 2018; Yamada et al., 2019).

In addition to the regulation of specific adsorbed species during electrochemistry, the subsequent interfacial evolution is extremely important. The successive interfacial reactions are mainly driven by electron transfer based on a radical reaction mechanism. Usually, the outer organic layer is vulnerable to attack due to the preferential propagation of free radicals at the interface between the outer interfacial phase layer and the electrolyte, leading to organic polymerization (Soto et al., 2015). The evolutionary origin of the interfacial layer is therefore largely dependent on the electrochemical stability of the outer organic components. It determines which component is polymerized first and to what extent the dissolution and growth of the interfacial layer can occur. In particular, for some carbon anode materials with special microstructures (e.g., mesopores or nanopores), inward growth may occur inside the material (Bommier et al., 2016). The interfacial phase growth is related to the electrochemical reactivity of the components in the matrix electrolyte in addition to the interfacial phase components. In addition, the transport of sodium ions in the interfacial layer is a key factor affecting the evolution of the interfacial layer growth. The migration of Na^+^ ions in the interfacial layer is related to the desolvation process associated with the solventization behavior, the migration of Na^+^ ions through the interfacial reaction products, and the crystallinity and composition distribution of the interfacial layer. Among them, the desolvation behavior of Na^+^ at the electrode/electrolyte interface is a key step in determining the reaction rate. The ion desolvation energy potential depends on a combination of factors such as the strength of ion-solvent or ion-ion interactions, the choice of electrode, the presence of interfacial membrane, and the composition or structural condition of the interfacial membrane (Yamada and Yamada, 2015; Yan et al., 2020).

### 4.3 Regulation strategy

**FIGURE 12:**
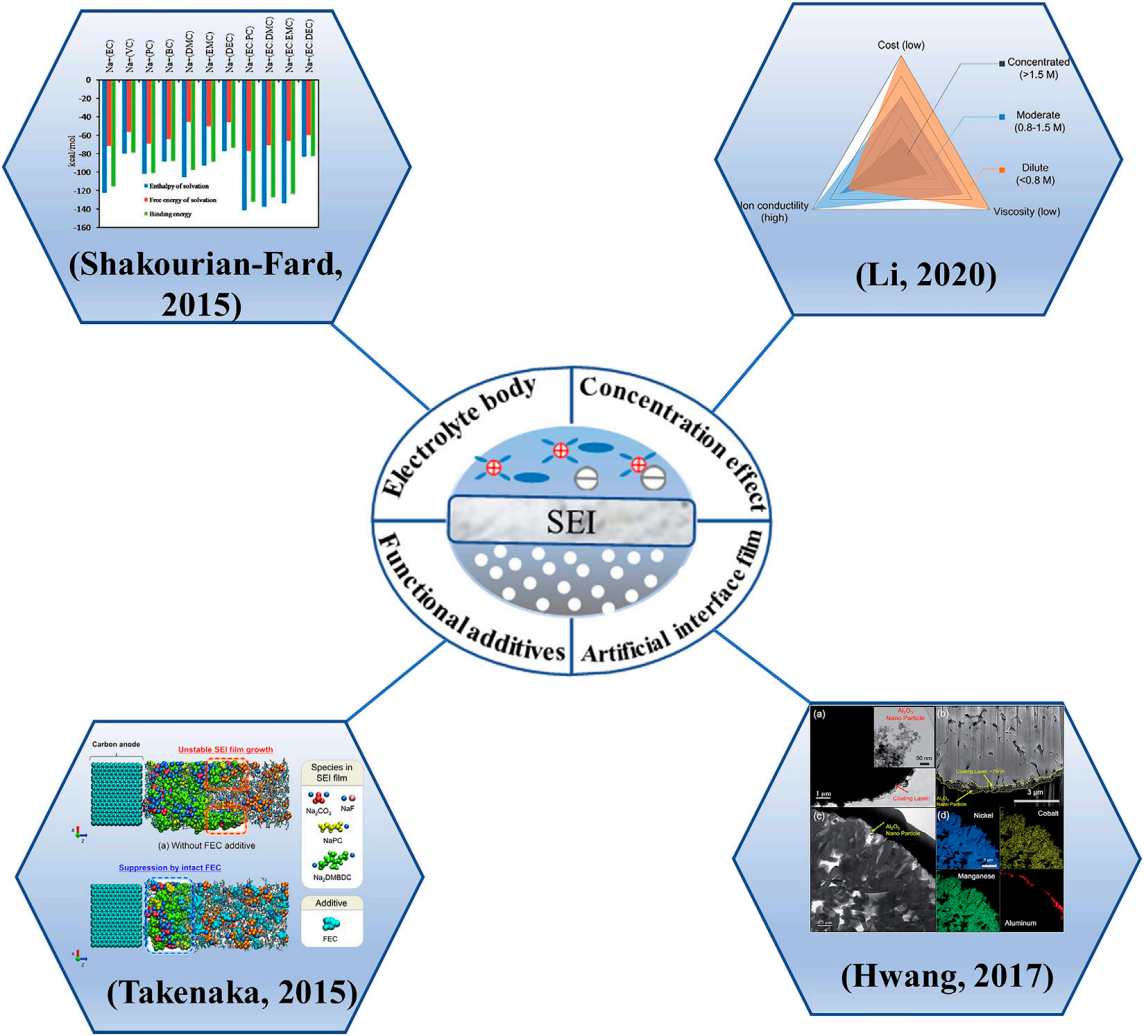
Modulation strategies for interfacial films.

At present, the regulation strategy for interfacial film mainly includes four parts: electrolyte body regulation, concentration regulation, addition of functional additives and construction of artificial interfacial film (Figure 12). They are described as follows.

#### 4.3.1 Electrolyte body

The actual state of the SEI actually depends on the choice of electrolyte composition, which determines to what extent the arrangement of inorganic and organic substances favors the final interfacial function. The current interfacial manipulation through the electrolyte ontology is mostly focused on the solventized structure of Na^+^, where the electrolyte solvent, salt anion, is involved (Hou et al., 2019), and a smaller percentage of external organic compounds by changing the solvent and sodium salt combination.

Solvents, as one of the participants in the solventized structure, mainly include esters, ethers and ionic liquids. For ester-based electrolytes, linear solvents can reduce the electrolyte viscosity and enhance the wettability, but usually linear solvents (DMC, DEC) are weakly coordinated with Na^+^ in the solventized structure, and therefore have high reactivity with the outer interfacial layer, which makes the interfacial layer unstable and may also increase the solubility of the interfacial layer (Xia et al., 2011; Ponrouch et al., 2013; Eshetu et al., 2016). The solventization of Na^+^ with cyclic EC and EC:PC is more favorable than that of linear solvents. Although the structural differences between EC and PC are small, the methyl group in PC may hinder the aggregation of reaction products from the perspective of long-term cell operation, resulting in insufficient formation of interphase layers (Takenaka and Nagaoka, 2019). In addition, since the LUMO level of Na^+^-solvent complexes decreases by 2–3 ev in varying degrees compared to a single solvent, the Na^+^-solvent complexes are more easily reduced on the anode surface and the increase in the HOMOLUMO energy band gap also leads to a longer operating window of the electrolyte. The addition of EC solvents to PC, DMC, EMC or DEC solvents results in the formation of a co-solvent, which reduces the band gap (Shakourian-Fard et al., 2015). Therefore, mixing different ratios of cyclic molecules (e.g., EC and PC) and chain molecules (DMC and DEC)) may lead to unexpected advantages. For ether solvents, such solvents can not only co-insert Na^+^ into graphite (Jache and Adelhelm, 2014; Kim et al., 2015; Cohn et al., 2016; Seidl et al., 2017), but also modulate the SEI passivation layer of the anode electrode material so that the interfacial phase composition has a better sodium ion transport rate and the generated passivation layer is thin and uniform (Soto et al., 2015; Wang C. et al., 2017; Zhang et al., 2017; He et al., 2018; Huang et al., 2019; Li et al., 2019). Among them, the special structure of amorphous Na_2_CO_3_ and NaF particles dispersed in polyether species improves the electrical conductivity of Na^+^ (Huang et al., 2019). However, the ether-derived interfacial phase is slightly lower than the ester-derived interfacial phase in terms of long-cycle performance. One optimizes the formation of the interfacial layer for high rate and long cycle performance by combining ester and ether, where the thick and loose ester-SEI is initially formed on the inside and the thin and dense ether-SEI is on the outside (Bai et al., 2018). ILs are actually salts in liquid state at room temperature compared to conventional molecular solvents (such as carbonates and ethers), which exhibit relatively low ionic conductivity due to strong interactions between their anions and cations, but they have the advantage of high electrochemical and thermal stability due to their low vapor pressure and low flammability. Therefore, hybrid electrolytes obtained by mixing ionic liquids with conventional molecular solvents such as organic carbonates can be used, thus combining all advantages to obtain interfacial layers with better performance (Monti et al., 2016; Manohar et al., 2018b).

Anionic salts, another major player in the solventized structure, play a crucial role in the oxidative decomposition of electrolytes along with the solvent. Some anions, such as BF_4_^−^ and PF_6_^−^, have been found to reduce the oxidative stability of common carbonate solvents, such as EC, PC and DMC, through fluorine or proton transfer reactions (Borodin and Jow, 2010). In addition, oxidation between the salt anion and the solvent through electrostatic interactions may occur accompanied by charge transfer phenomena to reach the final coupled state. This determines the oxidative stability of the solventized salt (Fadel et al., 2019). When Na^+^ is transported through the interfacial layer, anions with lower donor numbers (e.g., PF_6_^−^ and ClO_4_^−^) are more easily desolvated (Browning et al., 2017). Therefore, different anion-solvent complexes can have different effects on electrolyte oxidation stability during electrolyte decomposition on the cathode surface (Borodin and Jow, 2010; Xing et al., 2011; Cresce et al., 2015; Fadel et al., 2019). Different salts (e.g., NaClO_4_, NaFSI, NaTFSI) undergo different pathways when decomposed on the cathode surface, resulting in components with different properties that affect the performance of the interfacial layer. Therefore, mixed anions may provide additional benefits in regulating the interfacial chemistry.

#### 4.3.2 Concentration effect

The concentration effect is an important interfacial modulation strategy, and adjusting the optimized concentration can accordingly modulate the interfacial passivation chemistry to achieve an organic-inorganic equilibrium SEI layer. Traditionally, most electrolytes with optimized salt concentrations around 1 M exhibit the highest ionic conductivity (Yamada et al., 2019). Although the increase in salt concentration decreases the ionic conductivity, it exhibits special advantages in terms of enhanced interfacial properties and electrochemical behavior that conventional electrolytes do not possess. The decomposition order between solvent and salt differs with salt concentration. At conventional dilution concentrations the solvent decomposes preferentially, while at high concentrations, where there are almost no free solvent molecules left due to the urgent need to satisfy the dissolution of a large number of cations, the anion is forced to be decomposed first (Yamada and Yamada, 2015; 2017; Yamada et al., 2019; Yan et al., 2020). Li et al. (2020) found that, by reducing the sodium salt concentration (0.3 mol NaPF_6_/EC + PC (1:1 by volume)), the solvent molecules can fully occupy the Na^+^ solventized sheath layer, and the CEI and SEI films with high organic content (high C + O ratio) can be obtained on the cathode and anode sides. As the concentration of PF_6_^−^ is reduced, the decomposition by-products such as F, which has a corrosive effect on the electrode materials, are reduced, and the obtained SEI and CEI films are more stable. On the contrary, when applying high salt concentration electrolyte, the Na^+^ solvated sheath layer (or ligand layer) is almost occupied by anions. This will bring some special advantages. For example, the interfacial mass transfer process is changed and fast reactions can be performed on the electrode, the electrolyte volatility is weakened, the thermodynamic stability is enhanced and the safety is improved, the good SEI film can be formed on the electrode, and the Al collector is protected from anion corrosion. Increasing sodium salt concentration has an effect on the properties of the Na^+^ solubilization environment (Wahlers et al., 2016; Chen et al., 2018; Kankanamge et al., 2018; Li et al., 2018), charge transport mechanism (Forsyth et al., 2016; Kankanamge et al., 2018), and Na^+^ conductivity (Forsyth et al., 2016; Kankanamge et al., 2018). In addition, the overall ionic conductivity of the ether solvent-based electrolytes showed a tendency to increase with increasing sodium salt concentration. Electrolytes based on ionic liquid solvents promote ionic movement with the continuous addition of salts (Chen et al., 2018). Therefore, there are concepts of hyperconcentration and localized high concentration electrolytes have been proposed in recent years. Superconcentration because it promotes preferential passivation of LUMO-level reducing anions, which in turn creates a more powerful inorganic SEI that can better mitigate the more severe solubility problems of organic components. Moreover, a lower percentage of free solvent molecules will mitigate the tendency of soluble components to dissolve into the electrolyte (Takada et al., 2017). Localized high electrolyte concentrations not only do not alter the local dissolution environment of the concentrated electrolyte, but also provide the advantage of interface enhancement or suppression of undesirable interface problems (Zheng et al., 2018; Yamada et al., 2019).

#### 4.3.3 Functional additives

Additivity refers to the introduction of small doses of foreign molecules into the parent electrolyte. As one of the most economical interfacial modulation strategies, the addition of additives not only does not interfere with the overall properties of the electrolyte, but also significantly tunes the interfacial layer properties to better form films for interphase passivation for electrode protection and thus improve the overall electrochemical performance. Currently, FEC is the most widely reported and effective additive. Its energy gap, E_g_ is located between the HOMO-LUMO gap of salt and solvent, thus FEC can stabilize the interfacial layer by sacrificing decomposition in advance thus avoiding destructive decomposition of electrolyte (Wang et al., 2020). For example, the reason for the good performance in EC-based electrolytes containing FEC is that FEC has lower decomposition energy compared to EC solvents, and the presence of FEC also enhances the decomposition energy of EC molecules, so that its early decomposition in EC-based electrolytes promotes the generation of interfacial films (Kumar et al., 2016). In PC-based electrolytes, FEC also shows good film formation due to its still early decomposition compared to PC (Takenaka et al., 2015). It should be noted here that due to the strong electronegativity of F atoms, FEC attracts the positive charge of organic products, and excess FEC prevents the formation of dimers between organic monomer products, a phenomenon that causes undesired interfacial layer growth and adversely affects the stability of interfacial films (Takenaka et al., 2015; Simone et al., 2017; Bouibes et al., 2018). In addition to FEC, other additives as described above also contribute to the formation of stable interfacial films and need to be used in combination with different electrolyte and electrode systems for screening. In addition, the combined use of different additives may also be beneficial to achieve the desired stable interfacial film.

#### 4.3.4 Artificial interface film

Usually, the electrode itself is attacked by the highly reactive electrolyte, which may undergo structural changes or cause surface defects, *etc.* This will lead to further decomposition of the electrolyte, resulting in lower first-loop Coulomb efficiency and thicker interfacial films. Therefore, surface coating of the electrode can effectively improve its surface properties and improve the compatibility with the electrolyte. In fact, some of the cladding work done at the cathode is also equivalent to artificially creating CEI films. By using atomic layer deposition (ALD) to coat metal oxides (AlO_3_, TiO_2_ and MgO), metal fluorides and even solid electrolytes on the surface of the cathode material, these coatings maintain the stability of the reversible phase change of the cathode material during charging and discharging, or prevent the erosion of the cathode material by by-products such as HF, or provide better channels for Na^+^ transport provides a better channel and acts as a CEI film. For example, Sun et al. (Hwang et al., 2017a) changed the interfacial properties of the cathode by coating a layer of nano-Al_2_O_3_ on the surface of Na[Ni_0.6_Co_0.2_Mn_0.2_]O_2_ cathode. On the one hand, nano-Al_2_O_3_ can react with F present in the electrolyte to reduce the content of HF and prevent the continuous accumulation of NaF as a by-product to hinder Na^+^ conduction; on the other hand, AlF_3_, the product of nano-Al_2_O_3_ and HF, can enhance the protection of the cathode material by CEI film as a good component of CEI film. With the synergistic effect of the two, the change of interfacial impedance of the coated cathode material during the cycling process was significantly smaller than that before the coating, and the presence of the coating layer also helped to reduce the leaching of transition metals from the active material. Ye et al. (2022) reported an *in situ* artificial CEI construction strategy based on a spontaneous redox reaction between a pre-sodiumed organic solvent and a polyvinylidene fluoride (PVDF) binder. Applying this strategy to PB cathodes, the chemically pretreated PB cathodes were successfully coated with a NaF-rich interfacial phase on the electrode surface to keep them away from electrolyte attack and maintain cycling stability. This artificial CEI based on the interaction between PVDF and organic solvents is not much affected by the surface properties of the cathode material and is expected to be applied to other cathode materials. Therefore, the construction of artificial interfacial film is also an effective means to improve the compatibility of electrode with electrolyte and avoid some side reactions.

In summary, there are four common effective strategies for the regulation of solid electrolyte interfacial film. In practice, it is necessary to consider the characteristics of the electrolyte and electrode materials to choose the appropriate regulation. If the electrolyte body has a great influence on the interfacial membrane, it can try to use different salt and solvent mixing to regulate the interfacial membrane. If the electrolyte interfacial film is regulated on the basis of not changing the electrolyte body, the concentration of electrolyte or functional additives can be changed to achieve the purpose of regulating the appropriate interfacial film. If the interfacial film formed inside the battery system is unstable and cannot be improved by adjusting the electrolyte body, concentration, or additives, a suitable artificial interfacial film can be constructed from the electrodes to realize excellent electrochemical performance.

## 5 Conclusion and outlook

With the advantages of abundant sodium resources and low cost, sodium ion batteries are a promising energy storage battery system. At present, researchers at home and abroad have developed a variety of feasible cathode and anode materials for sodium ion batteries. The electrolyte of sodium ion battery, as a medium for the cathode and anode materials to participate in the redox reaction, has an important influence on the thermodynamic and kinetic properties of the sodium ion battery system, such as the structural stability of the electrode materials, the composition and structure of the SEI, the multiplicative performance, cycling stability and thermal stability of the battery. Therefore, electrolyte is also the key to determine the battery performance. This paper introduces the research progress of organic liquid electrolytes for sodium ion batteries from the basic requirements and composition of organic liquid electrolytes, the current research status of organic liquid electrolytes, and the composition, requirements and regulation strategies of the interface between electrolytes and electrodes. First, an overview of organic liquid electrolytes is introduced, followed by the classification of organic liquid electrolytes from the perspective of sodium salts, and the compatibility and electrochemical properties of each sodium salt electrolyte with cathode and anode are introduced. Finally, the strategy for electrolyte regulation of interfacial film is explained. At present, organic liquid electrolytes for sodium ion batteries still have problems such as narrow electrochemical windows and poor stability of SEI films. The development of new, low-cost and high-performance sodium ion battery electrolytes is crucial for the commercialization of sodium ion batteries. Future research on organic liquid electrolytes for sodium ion batteries can be carried out from the following aspects.

(1) Optimization of each individual component of the organic liquid electrolyte, including its own physical and chemical properties such as viscosity, conductivity, stability, *etc.* The compatibility of the electrolyte with the electrode material is also crucial. For example, the commonly used ester electrolyte cannot be applied to graphite anode materials, but the ether electrolyte allows sodium ions to enter the interlayer energy storage in a solventized form. The selection of additives also needs to consider the compatibility with electrolyte and electrode materials as well. In addition, in the future, we should try to explore the internal energy balance of solvent molecules from the perspective of molecular dynamics simulation and analyze the sodium storage mechanism of sodium ion battery in combination with the special structure of electrode materials, which is more conducive to enhance the matching of electrolyte and electrode materials to achieve high capacity requirements. In conclusion, we should focus on the matching of electrode electrolyte and the development of new sodium salts and additives to achieve high performance of sodium ion batteries.
(2) Battery safety is the most important key indicator of market and customer concern. Commonly used organic electrolytes cannot operate properly in high temperature environments, so the use of stable Na salts and non-flammable solvents including ionic liquids and phosphate esters to replace traditional flammable solvents, as well as the addition of flame retardant additives and overcharge additives are within consideration to achieve sodium ion battery safety. In general, the use of stable Na salts increases the thermal stability of the electrolyte, and high concentrations of the electrolyte exhibit reduced flammability. The use of non-flammable solvents instead of traditional flammable solvents is attractive because they can make the electrolyte completely non-flammable. Electrolyte reformulation consisting of flame retardant additives and overcharge additives is economical and effective. However, the high cost required for nonflammable electrolytes is a significant limitation to their commercialization.
(3) The electrolyte of sodium ion battery, as a medium for the cathode and anode materials to participate in the redox reaction, its redox window, the migration and diffusion of sodium ions, the solventized structure of sodium ions, and the coupling correlation effect between sodium ions and anions or solvents are the key factors that determine the interfacial properties of electrode materials. In addition, the sensitive nature of the interfacial phase increases the difficulty of characterization and limits our understanding of the interfacial phase. Regulation of electrolyte proprieties, concentration effects, electrolyte additives, and artificial interfacial films are effective methods to manipulate interphase formation. To meet the requirements of applications, the enhancement of interfacial composition, structure, and stability requires more fundamental work, theoretical computational studies, and advanced testing and analysis methods.

In conclusion, in the context of the imminent commercialization of sodium-ion batteries, substantial progress has been made in the research on positive and negative electrode materials. For example, the anode materials currently used in commercialized sodium-ion batteries are all hard carbon. There are already examples of commercialized production of the three main types of cathode materials. However, the organic electrolyte system used is still the solvents (EC:PC, EC:DEC or PC as a single solvent) and the sodium salts (NaClO_4_ and NaPF_6_). There is still a long way to go for the commercialization of organic liquid electrolytes corresponding to specific scenarios (high voltage, wide temperature, non-flammable). In the future, for the development of organic liquid electrolytes, great efforts are still needed to design safer electrolytes and more stable interfaces for SIBs. The optimization of electrolyte and solid electrolyte interface films will further bring sodium ion batteries closer to practical applications, allowing them to be widely used in the direction of large-scale energy storage and promoting commercial applications.
